# Burden, trends, and projections of nutritional deficiencies in China from 1990 to 2030

**DOI:** 10.3389/fnut.2025.1643869

**Published:** 2025-09-04

**Authors:** Bijuan Chen, Huanhuan Yang, Wei Zheng, Hanchen Zheng, Hui Lin, Jiami Yu, Yun Xu, Zengqing Guo, Zhouwei Zhan

**Affiliations:** ^1^Department of Radiation Oncology, Clinical Oncology School of Fujian Medical University, Fujian Cancer Hospital, Fuzhou, China; ^2^Department of Medical Oncology, Clinical Oncology School of Fujian Medical University, Fujian Cancer Hospital, Fuzhou, China

**Keywords:** nutritional deficiencies, China, global burden of disease, age-period-cohort analysis, epidemiology, projection

## Abstract

**Background:**

Nutritional deficiencies remain a pressing public health concern, especially in countries undergoing rapid demographic and epidemiologic transitions. In China, understanding the long-term trends and sex- and age-specific burden of nutritional deficiencies is crucial for designing targeted prevention and intervention strategies.

**Methods:**

We used data from the Global Burden of Disease Study 2021 to estimate the burden of nutritional deficiencies in China from 1990 to 2021. Indicators included incidence, prevalence, mortality, disability-adjusted life years (DALYs), years lived with disability (YLDs), and years of life lost (YLLs), stratified by age, sex, and type of malnutrition. We applied Joinpoint regression to examine temporal trends and conducted age-period-cohort (APC) and decomposition analyses to explore underlying drivers. Projections through 2030 were generated using Bayesian APC modeling.

**Results:**

In 2021, nutritional deficiencies led to 46.0 million incident and 146.1 million prevalent cases in China, with a significantly higher burden among women. While males had higher mortality and YLL rates, females showed higher prevalence, YLDs, and DALYs. The age-standardized burden declined substantially from 1990 to 2021, particularly for protein-energy malnutrition. DALYs declined by 92.8% for protein-energy malnutrition, and vitamin A deficiency incidence dropped by 81.2%. APC analysis revealed that younger cohorts experienced dramatically lower burdens, especially among children under 5, although older adults continued to carry a growing burden due to aging. Decomposition analysis identified epidemiologic improvements as the primary driver of reduced burden, but demographic factors like population aging mitigated these gains. Forecasts indicate further declines in disease burden through 2030, with consistently higher prevalence and DALY rates projected among females.

**Conclusion:**

Despite substantial progress in reducing the burden of nutritional deficiencies in China over the past three decades, disparities persist by sex and age. Continued surveillance, alongside interventions targeting women and older adults, is essential to sustain progress and address residual gaps.

## Introduction

Nutritional deficiencies remain a major public health concern worldwide, contributing significantly to the global burden of disease (1, 2). Despite overall progress in economic development and healthcare systems, malnutrition continues to affect populations in both low- and middle-income countries and increasingly in aging societies (3, 4). The Global Burden of Disease (GBD) study has systematically evaluated the health impacts of nutritional deficiencies, revealing substantial morbidity and mortality, particularly among vulnerable groups such as children, women, and the elderly (5). Epidemiological evidence has linked nutritional deficiencies to a wide range of adverse health outcomes. For example, protein-energy malnutrition significantly increases the risk of in-hospital mortality (odds ratio [OR]: 2.00) (6), while iron deficiency anemia has been associated with an estimated global burden of 600,000 perinatal and 100,000 maternal deaths per year (7). Furthermore, vitamin A deficiency has been shown to raise susceptibility to infections and increase childhood mortality (8). These associations underscore the systemic health consequences of malnutrition and reinforce the need for continuous surveillance and prevention. These deficiencies not only impair physical development and immune function but also increase the risk of noncommunicable diseases (NCDs) and infectious diseases, posing long-term challenges to public health systems (9, 10). The complexity of malnutrition includes both undernutrition and micronutrient deficiencies, which require targeted surveillance and policy attention to support prevention, early detection, and sustainable intervention strategies.

In China, the epidemiological transition over the past three decades has led to shifting patterns in nutritional deficiency-related disease burden. Rapid socioeconomic development, urbanization, and changes in dietary habits have influenced health behaviors and outcomes across different population subgroups (11, 12). For instance, a recent study by Zhang et al. analyzed trends in child undernutrition from 2000 to 2019 using national burden of disease data, highlighting persistent provincial disparities and a slowdown in progress after 2010 (13). While valuable, such studies are largely focused on children under five and limited to a subset of indicators, such as stunting, wasting, and underweight. Studies have shown that although overall rates of malnutrition have declined, the age-standardized burden remains substantial in specific subpopulations, particularly among the very young and elderly, with regional and sex-based disparities (14, 15). However, comprehensive national assessments spanning multiple nutritional deficiency subtypes (e.g., protein-energy malnutrition, iron deficiency, vitamin A deficiency) and broader age groups remain scarce. Additionally, trends in incidence, prevalence, and disability-adjusted life years (DALYs) attributable to malnutrition reflect the interaction of demographic aging, healthcare access, and policy implementation. Our study addresses this gap by leveraging GBD 2021 data to evaluate long-term and projected trends across nutritional deficiency types, stratified by age, sex, and region. Understanding these patterns is essential for informing public health policy and resource allocation, particularly in the context of China’s aging population and emerging double burden of malnutrition.

Despite extensive progress in reducing absolute numbers, the persistent and uneven burden of nutritional deficiencies necessitates more nuanced, disaggregated analysis. Previous literature has largely focused on specific nutrient deficiencies (e.g., protein-energy malnutrition, iron, vitamin A) (16–18), while limited attention has been paid to comprehensive temporal trends stratified by sex and age over the long term. Moreover, disparities in disease burden between sexes, shaped by a complex interplay of biological, sociocultural, and healthcare access factors, remain inadequately investigated. A deeper understanding of age-period-cohort effects can further elucidate how generational exposures and historical context shape disease burden across life stages. Against this background, our study utilizes the most recent GBD 2021 data to analyze the long-term trends and projections of nutritional deficiency burden in China from 1990 to 2021, disaggregated by sex, age group, and type of malnutrition, thereby providing crucial evidence for targeted interventions and sustainable policy-making.

## Methods

### Data source

This study is based on publicly available data from the GBD 2021, conducted by the Institute for Health Metrics and Evaluation (IHME). The GBD 2021 provides comprehensive and comparable estimates of disease burden across 204 countries and territories, spanning from 1990 to 2021. It includes estimates for incidence, prevalence, mortality, DALYs, years lived with disability (YLDs), and years of life lost (YLLs) for 371 diseases and injuries, as well as 88 risk factors (19, 20). All data specific to China were retrieved from the Global Health Data Exchange (GHDx) platform,^1^ with a focus on indicators associated with nutritional deficiencies, including incidence, prevalence, mortality, DALYs, YLDs, and YLLs. The estimates were generated using the DisMod-MR 2.1 modeling framework, which synthesizes data from multiple sources including epidemiological studies, household surveys, national censuses, hospital and clinical records, disease registries, and vital registration systems. These inputs are adjusted for measurement errors and standardized for comparability across locations and time periods (19, 20). Age-standardized rates were calculated using the GBD world standard population to account for variations in population age structure, allowing for accurate temporal and intergroup comparisons. Only data from 1990 to 2021 for China were included in the present analysis. Data were stratified by age, sex, calendar year, and malnutrition subtype to enable detailed trend analysis and modeling.

### Definition and estimation

In this study, nutritional deficiencies were defined according to the classification framework established by the GBD 2021. Nutritional deficiencies comprise mortality and disability associated with five primary categories: protein-energy malnutrition, vitamin A deficiency, dietary iron deficiency, iodine deficiency, and other nutritional deficiencies (19, 20). Protein-energy malnutrition refers to health loss resulting from moderate or severe acute wasting, defined using anthropometric indicators such as weight-for-height z-score (WHZ) below −2 standard deviations or a mid-upper arm circumference (MUAC) less than 125 mm, in accordance with WHO guidelines. Within the GBD 2021 framework, protein-energy malnutrition is further disaggregated into four clinical subtypes: moderate wasting with or without oedema, and severe wasting with or without oedema, which reflect increasing severity and are modeled separately using standardized case definitions based on WHZ and/or MUAC thresholds (18, 20). Vitamin A deficiency is defined as serum retinol concentration less than 0.70 μmol/L and includes disabilities attributable to blindness, vision loss, and anemia caused by vitamin A deficiency (21). Dietary iron deficiency is defined solely as inadequate intake of elemental iron leading to anemia. This definition excludes iron loss due to conditions such as parasitic infections or gastrointestinal bleeding, which are attributed to their underlying causes (22). Iron deficiency without anemia is not included in the estimates. Iodine deficiency captures outcomes associated with insufficient iodine intake, including goiter, hypothyroidism, and intellectual disabilities in children (23). Other nutritional deficiencies encompass less common micronutrient insufficiencies such as vitamin D, vitamin C, folate, niacin, thiamine, calcium, and selenium deficiencies, which are not modeled independently but grouped together due to data limitations (24). For each category, GBD 2021 estimated the incidence, prevalence, deaths, and disability burden using a Bayesian meta-regression tool, DisMod-MR 2.1, which ensures consistency between epidemiological parameters and accounts for variations across age, sex, year, and location. Uncertainty intervals (UIs) were calculated based on 1,000 posterior simulations and reported as the 2.5th and 97.5th percentiles (20).

### Descriptive analysis

We conducted a descriptive analysis to examine the burden of nutritional deficiency-related diseases in China from 1990 to 2021, using indicators including incidence, prevalence, mortality, DALYs, YLDs, and YLLs. Analyses were stratified by sex, age group, calendar year, and malnutrition subtype. Both absolute numbers and age-standardized rates were extracted, with standardization based on the GBD global reference population to enable temporal comparisons. We visualized trends using line charts to illustrate long-term changes and demographic disparities, particularly focusing on high-burden groups such as young children and older adults. Sex-specific patterns were also assessed to explore gender differences in disease burden. Temporal comparisons highlighted shifts driven by epidemiological and demographic transitions. All analyses were performed using R software (version 4.3.1), and 95% UIs were calculated from 1,000 posterior draws based on the GBD estimation framework.

### Joinpoint regression analysis

Joinpoint regression analysis was employed to quantify temporal patterns in age-standardized rates of total and distinct subtypes of nutritional deficiency-related burden indicators, including incidence, prevalence, mortality, DALYs, YLDs, and YLLs, over the period from 1990 to 2021. This method identifies points in time (joinpoints) at which statistically significant changes in trend occur, and fits a series of linear segments connected at these joinpoints to model the annual percentage change (APC) for each segment and the average annual percentage change (AAPC) over the entire period. Statistical significance is assessed using Monte Carlo permutation tests, which evaluate whether the inclusion of additional joinpoints significantly improves model fit at a 0.05 significance level. We applied the Joinpoint Regression Program (version 5.2.0) developed by the US National Cancer Institute. A maximum of five joinpoints was allowed for each model, and the optimal number was selected using the Monte Carlo permutation method. Significance was defined as *p* < 0.05. Separate models were constructed for each sex and for each indicator. This analysis enabled us to detect important shifts in disease burden trends, providing insights into the timing and direction of changes across different nutritional deficiency subtypes.

### Age-period-cohort analysis

To disentangle the temporal effects of age, period, and birth cohort on the trends in incidence, prevalence, and DALYs related to nutritional deficiencies, we conducted an APC analysis. This method helps assess how different generations and time periods contribute to the disease burden, while adjusting for age-related physiological and demographic changes. We categorized data into consecutive 5-year age groups (from <5 to 95+ years), 5-year calendar periods (1992–1996, 1997–2001, etc.), and corresponding birth cohorts. The classic APC model was fitted using the intrinsic estimator (IE) method, which resolves the identifiability problem among age, period, and cohort effects by imposing a constraint that provides unbiased and consistent estimates (25, 26). This analysis was performed using the Epi package (version 2.51) in R (version 4.3.1), which allows estimation of age, period, and cohort effects through the intrinsic estimator method. The APC results provided valuable insights into generational shifts in disease burden, highlighting specific age groups and cohorts that might benefit from targeted nutritional interventions.

### Decomposition analysis

To determine the key drivers behind the changes in nutritional deficiency-related disease burden in China from 1990 to 2021, we performed a decomposition analysis. This method separates the absolute change in crude incidence, prevalence, and DALYs into three contributing factors: population growth, population aging, and epidemiological changes. This framework enables a clearer understanding of how demographic and health-related transitions have influenced overall burden trends over time. Following the approach proposed by Das Gupta, we calculated the contributions of each factor by comparing observed values in 2021 with counterfactual scenarios where one component (e.g., age-specific rates, age structure, or total population size) is held constant while the others are updated. Specifically, population growth reflects the effect of an increase in the total number of people, population aging captures shifts in the population’s age composition, and epidemiological changes represent changes in age-specific rates due to prevention, diagnosis, treatment, or broader health system improvements (27, 28). This method allows for the quantification of each component’s isolated impact without requiring explicit mathematical formulas in the final output. All analyses were conducted using R software (version 4.3.1). The decomposition analysis was performed using custom R scripts based on manually written mathematical formulas to quantify the contributions of population aging, population growth, and changes in age-specific rates. Visualizations were generated with the ggplot2 package for clear presentation of the results.

### Bayesian age-period-cohort analysis

To project future trends in the burden of nutritional deficiencies, we employed a BAPC model, which allows for robust and probabilistic estimations of future incidence, prevalence, and DALYs by sex. This model captures the temporal dynamics of disease burden by disentangling the effects of age, calendar period, and birth cohort while incorporating uncertainty in a Bayesian framework. The analysis was conducted using the R package BAPC, which utilizes Integrated Nested Laplace Approximation (INLA) for computational efficiency and accurate estimation of posterior distributions. We used historical age-specific rates from 1990 to 2021 as input and generated projections through 2030. The model assumes second-order random walks (RW2) for the age, period, and cohort effects to smooth temporal fluctuations while preserving structural trends. By generating 95% UIs around the projected estimates, the BAPC model offers a nuanced understanding of how the disease burden may evolve, aiding long-term public health planning and resource allocation.

### Ethics approval

This study used publicly available, de-identified GBD data; therefore, ethical approval and informed consent were not required. The study was conducted in compliance with the Guidelines for Accurate and Transparent Health Estimates Reporting (GATHER) (29).

## Results

### Burden of nutritional deficiencies in China, 2021

In 2021, nutritional deficiencies remained a substantial public health concern in China. The total number of all-age incident cases reached approximately 46.0 million, while prevalent cases climbed to 146.1 million. Notably, women bore a higher burden in prevalence than men, with 93.3 million cases compared to 52.9 million. Correspondingly, the age-standardized incidence rate was 3408.88 per 100,000 population, with a slightly higher rate in females (3502.24) than males (3332.08). The age-standardized prevalence rate was significantly greater in females (12827.81) than in males (7198.86), reflecting gender disparities in nutritional vulnerability. There were 15,756 deaths attributed to nutritional deficiencies, with an age-standardized death rate of 1.14 per 100,000; this rate was nearly twice as high in males (1.67) as in females (0.86). The overall DALYs amounted to 2.3 million, translating to an age-standardized DALY rate of 159.31 per 100,000. Females experienced a much higher burden (231.69) compared to males (93.45), driven primarily by YLDs rather than YLLs. YLDs were the dominant component, accounting for 2.0 million of the total DALYs, while YLLs contributed 265,735 years. These metrics highlight the considerable health burden posed by nutritional deficiencies in China, especially among women (Table 1).

**Table 1:** All-age cases and age-standardized incidence, prevalence, deaths, DALYs, YLDs, and YLLs rates in 2021 for nutritional deficiencies in China.

Measure	All-ages cases			Age-standardized rates per 100,000 people		
	Total	Male	Female	Total	Male	Female
Incidence	46,003,560 (40,820,797, 51,637,763)	23,378,949 (20,211,079, 27,025,070)	22,624,611 (19,795,078, 25,841,662)	3408.88 (3026.97, 3830.22)	3332.08 (2853.76, 3922.28)	3502.24 (3033.40, 4027.66)
Prevalence	146,147,146 (138,504,726, 154,667,650)	52,855,385 (49,212,139, 57,219,743)	93,291,761 (88,464,336, 98,479,131)	9890.92 (9410.55, 10463.85)	7198.86 (6680.69, 7824.98)	12827.81 (12160.04, 13529.31)
Deaths	15,756 (13,053, 18,627)	8,513 (7,048, 10,350)	7,243 (5,515, 9,079)	1.14 (0.94, 1.34)	1.67 (1.39, 2.00)	0.86 (0.66, 1.06)
DALYs	2,299,911 (1,564,018, 3,318,673)	599,825 (430,488, 841,180)	1,700,086 (1,137,998, 2,479,321)	159.31 (109.39, 226.47)	93.45 (69.26, 126.60)	231.69 (154.52, 335.43)
YLDs	2,034,175 (1,303,806, 3,063,407)	445,563 (277,437, 696,874)	1,588,612 (1,028,816, 2,363,992)	137.27 (88.18, 205.67)	64.42 (40.37, 98.83)	214.57 (138.74, 318.98)
YLLs	265,735 (222,098, 310,344)	154,262 (127,967, 189,467)	111,474 (88,873, 136,744)	22.04 (18.66, 25.66)	29.02 (24.52, 34.48)	17.12 (14.04, 20.78)

Values in parentheses indicate 95% UIs, estimated using Monte Carlo simulations. DALYs, disability-adjusted life-years; YLDs, years lived with disability; YLLs, years of life lost.

### Age- and sex-specific burden of nutritional deficiencies in China, 2021

**Figure 1:**
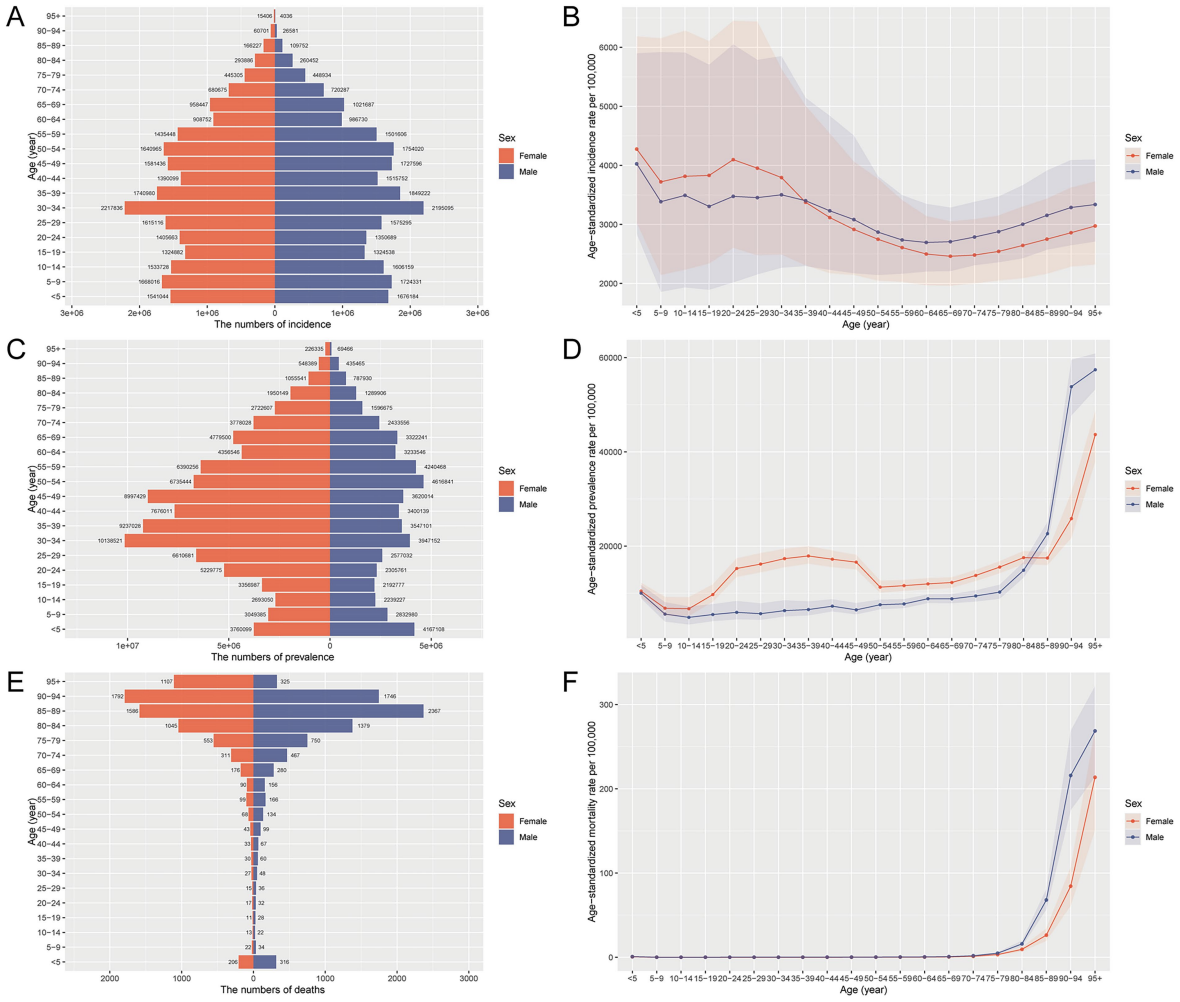
Age- and sex-specific number and age-standardized rates of incidence, prevalence, and mortality for nutritional deficiencies in China, 2021. **(A)** Number of incident cases by age and sex. **(B)** Age-standardized incidence rate per 100,000 population by age and sex. **(C)** Number of prevalent cases by age and sex. **(D)** Age-standardized prevalence rate per 100,000 population by age and sex. **(E)** Number of deaths by age and sex. **(F)** Age-standardized mortality rate per 100,000 population by age and sex.

In 2021, nutritional deficiencies in China demonstrated clear age- and sex-specific patterns in incidence, prevalence, mortality, and related health loss. As shown in Figure 1, the number of incident and prevalent cases was highest among children under 5 years, gradually declining in adolescence and middle age, before increasing again in the elderly population. Females exhibited slightly higher incident and prevalent case counts than males in younger and middle-aged groups, while males surpassed females in older age groups. The age-standardized incidence and prevalence rates were notably elevated in early childhood and among adults aged 70 years and above. Mortality burden showed a contrasting pattern, with deaths and age-standardized mortality rates increasing steeply after age 60, particularly among men, indicating greater vulnerability in older males. Supplementary Figure S1 further reveals that the overall burden of disease, measured by DALYs, was highest in children under 5 and adults over 70. YLDs were consistently higher in females across most age groups, reflecting prolonged periods of disability, while YLLs rose markedly in elderly men, indicating premature mortality as the dominant contributor to DALYs in that group. These findings underscore a bimodal distribution of nutritional burden in China, affecting both pediatric and geriatric populations with notable sex disparities.

### Age- and sex-specific trends in the burden of nutritional deficiencies in China, 1990–2021

**Figure 2:**
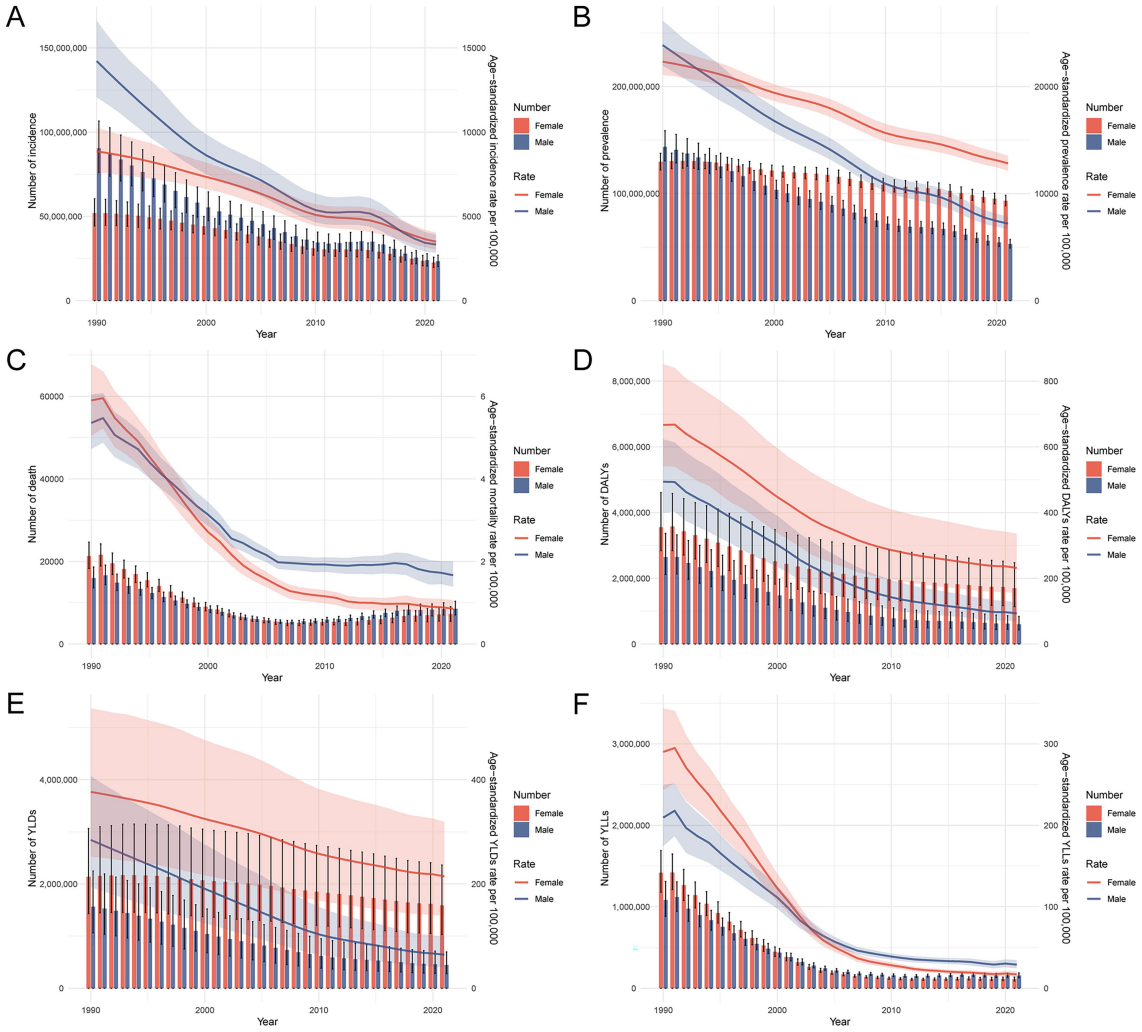
Trends in the number and age-standardized rates of prevalence, incidence, mortality, DALYs, YLDs, and YLLs for nutritional deficiencies in China by sex, 1990–2021. **(A)** Number and age-standardized prevalence rate per 100,000 population by sex. **(B)** Number and age-standardized incidence rate per 100,000 population by sex. **(C)** Number and age-standardized mortality rate per 100,000 population by sex. **(D)** Number and age-standardized DALYs rate per 100,000 population by sex. **(E)** Number and age-standardized YLDs rate per 100,000 population by sex. **(F)** Number and age-standardized YLLs rate per 100,000 population by sex. Abbreviations: DALYs, disability-adjusted life years; YLDs, years lived with disability; YLLs, years of life lost.

Between 1990 and 2021, the burden of nutritional deficiency-related diseases in China declined substantially across both sexes and all indicators (Figure 2). Age-standardized incidence, prevalence, mortality, DALYs, YLDs, and YLLs all decreased in both males and females. While males showed a more significant decline in incidence and mortality rates, females consistently exhibited higher YLDs and DALYs, reflecting a greater chronic burden. Mortality and YLL rates remained higher in males throughout the study period, while the prevalence rate surpassed that of males among females in later years. Supplementary Figure S2 further illustrates age-specific patterns in burden. Both the number and crude rates of incidence, prevalence, deaths, DALYs, YLDs, and YLLs declined significantly across all age groups. However, the distribution was uneven, with both infants and older adults consistently experiencing the greatest burden, forming a characteristic U-shaped pattern across age groups. For example, children under five showed over 80% reductions in incidence, DALYs, and deaths, yet still accounted for the highest burden in 2021. Older adults (especially 85+ years) exhibited relatively stable or slower declines in rates, likely due to population aging and chronic disease accumulation. These findings underscore the ongoing need for targeted nutritional interventions among the most vulnerable age groups at both ends of the lifespan.

### Trends in the burden of different types of nutritional deficiencies in China, 1990–2021

**Figure 3:**
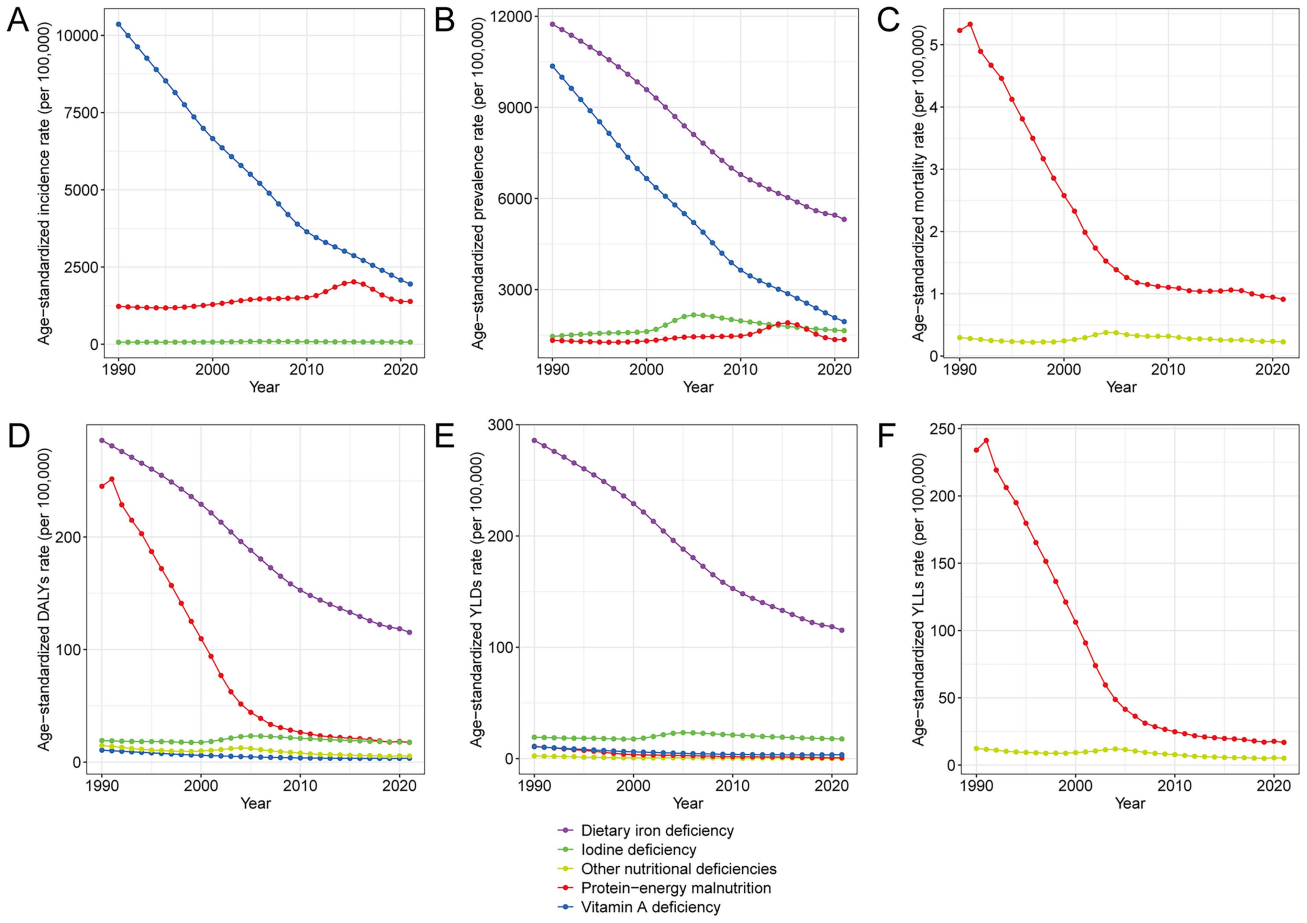
Trends in age-standardized rates of disease burden attributable to different types of malnutrition in China, 1990–2021. **(A)** Age-standardized incidence rate per 100,000 population by malnutrition type. **(B)** Age-standardized prevalence rate per 100,000 population by malnutrition type. **(C)** Age-standardized mortality rate per 100,000 population by malnutrition type. **(D)** Age-standardized DALY rate per 100,000 population by malnutrition type. **(E)** Age-standardized YLD rate per 100,000 population by malnutrition type. **(F)** Age-standardized YLL rate per 100,000 population by malnutrition type. Abbreviations: DALYs, disability-adjusted life years; YLDs, years lived with disability; YLLs, years of life lost.

Figure 3 and Supplementary Tables S1, S2 illustrate the long-term trends in disease burden attributable to various types of nutritional deficiencies in China from 1990 to 2021. Across all key metrics, including incidence, prevalence, mortality, DALYs, YLDs, and YLLs, protein-energy malnutrition exhibited a substantial decline in age-standardized mortality (82.6%) and DALYs (92.8%). These improvements occurred despite modest rises in incidence and prevalence, suggesting notable progress in survival outcomes and the management of related disabilities. Vitamin A deficiency also showed substantial decreases in both incidence and DALY rates (−81.2% and −67.7%, respectively), while dietary iron deficiency maintained the highest prevalence in 2021 (5313.2/100,000), though with a 54.7% decline from 1990. Iodine deficiency showed a more modest reduction in burden, with only a −7.8% change in DALY rates. Mortality and YLLs were primarily concentrated in protein-energy malnutrition, while YLDs were notably high for dietary iron deficiency and iodine deficiency, suggesting a shift from fatal to chronic disabling outcomes. Overall, the burden of malnutrition in China has significantly declined over the past three decades, but certain deficiencies, particularly iron and iodine-related disorders, continue to contribute to a substantial public health burden, warranting targeted nutritional interventions.

### Trends in nutritional deficiency burden in China compared to global levels, 1990–2021

Supplementary Figure S3 and Table 2 highlight comparative changes in the burden of nutritional deficiencies between China and the global average from 1990 to 2021. Over this period, China demonstrated significantly greater reductions in all age-standardized indicators of disease burden than the global average. Notably, the AAPC in age-standardized incidence and prevalence rates in China showed declines of −3.97% and −2.73% per 100,000, respectively, compared to global declines of −2.53% and −0.96%. Mortality and DALYs in China also decreased more markedly (AAPC: −4.97% and −4.15%) than the global declines (−3.84% and −2.28%), indicating improved survival and overall health outcomes. YLDs and YLLs experienced substantial reductions in China (AAPC: −2.78% and −7.68%) versus more modest global declines (−0.86% and −4.67%), underscoring enhanced chronic disease management and decreased premature mortality. Supplementary Figure S3 visually reinforces these disparities, showing that the pace of decline across all burden indicators is steeper in China than globally. These findings reflect the effectiveness of China’s national nutritional policies and public health interventions over recent decades, positioning it as a leading example in malnutrition control efforts.

**Table 2:** Change of age-standardized rates in incidence, prevalence, deaths, DALYs, YLDs, and YLLs for nutritional deficiencies between 1990 and 2021 in China and global level.

Measure	China			Global		
	1990	2021	AAPC	1990	2021	AAPC
Incidence	11654.93 (10317.82, 13127.84)	3408.88 (3026.97, 3830.22)	−3.97 (−4.03 to −3.92)^*^	17112.55 (16470.31, 17731.43)	7725.1 (7404.01, 8109.01)	−2.53 (−2.54 to −2.52)^*^
Prevalence	23093.68 (21856.90, 24477.24)	9890.92 (9410.55, 10463.85)	−2.73 (−2.75 to −2.71) ^*^	32217.95 (31693.55, 32740.92)	23858.99 (23445.77, 24320.82)	−0.96 (−0.97 to −0.95)^*^
Deaths	5.53 (4.85, 6.19)	1.14 (0.94, 1.34)	−4.97 (−5.09 to −4.86)^*^	10.9 (9.44, 12.97)	3.03 (2.69, 3.40)	−3.84 (−4.29 to −3.23)^*^
DALYs	575.41 (467.01, 729.98)	159.31 (109.39, 226.47)	−4.15 (−4.21 to −4.08) ^*^	1367.15 (1126.30, 1708.49)	657.62 (489.93, 869.58)	−2.28 (−2.66 to −1.94)^*^
YLDs	329.02 (223.20, 465.89)	137.27 (88.18, 205.67)	−2.78 (−2.80 to −2.77)^*^	664.12 (453.17, 933.22)	507.94 (343.56, 726.80)	−0.86 (−0.87 to −0.85)^*^
YLLs	246.4 (209.23, 289.81)	22.04 (18.66, 25.66)	−7.68 (−7.81 to −7.54)^*^	703.03 (589.12, 872.28)	149.68 (122.99, 178.12)	−4.67 (−5.18 to −4.00)^*^

Values in parentheses indicate 95% UIs, estimated using Monte Carlo simulations. DALYs, disability-adjusted life-years; YLDs, years lived with disability; YLLs, years of life lost; AAPC, average annual percent change. ^*^p < 0.05 (permutation test).

### Trends in nutritional deficiency burden in China by sex based on joinpoint regression analysis

**Figure 4:**
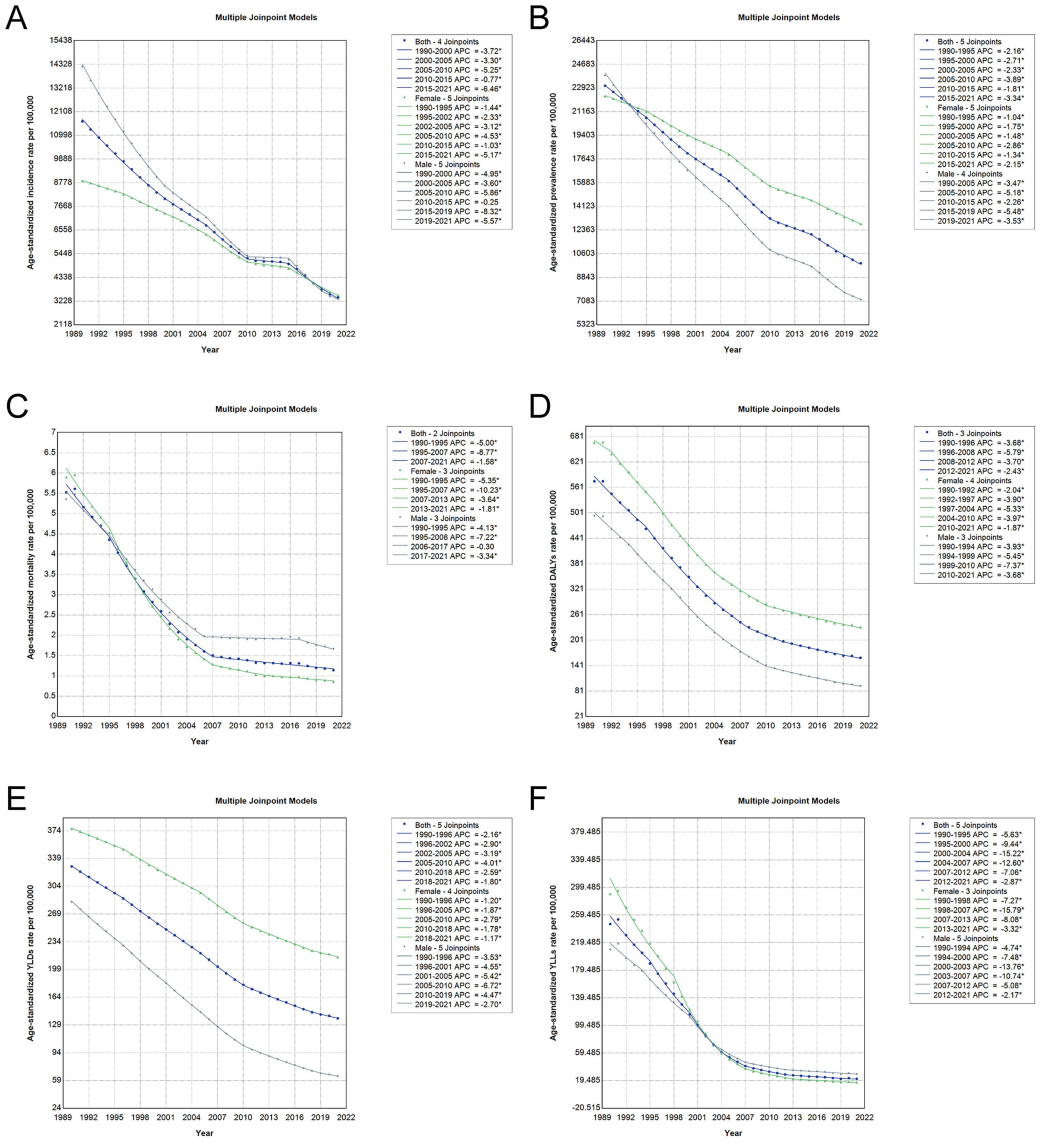
Joinpoint regression analysis of trends in age-standardized rates of malnutrition burden in China, 1990–2021. **(A)** Age-standardized incidence rate per 100,000 population. **(B)** Age-standardized prevalence rate per 100,000 population. **(C)** Age-standardized mortality rate per 100,000 population. **(D)** Age-standardized DALY rate per 100,000 population. **(E)** Age-standardized YLD rate per 100,000 population. **(F)** Age-standardized YLL rate per 100,000 population. Each panel shows the annual percentage change (APC) segments and corresponding joinpoints identified through Joinpoint regression. Abbreviations: DALYs, disability-adjusted life years; YLDs, years lived with disability; YLLs, years of life lost.

Joinpoint regression analyses presented in Figure 4 and Supplementary Tables S3, S4 reveal marked sex-specific temporal trends in the burden of nutritional deficiencies in China from 1990 to 2021. Overall, age-standardized incidence, prevalence, and mortality rates showed consistent declines in both sexes, with more pronounced reductions in males. Males experienced a greater annual decrease in age-standardized incidence rate (AAPC: −4.62%) compared to females (AAPC: −2.97%). In contrast, age-standardized mortality rate decreased faster in women (AAPC: −6.06%) than in men (AAPC: −3.81%), particularly during the 1990s and early 2000s. Trends in DALYs, YLDs, and YLLs were also downward across all periods. Notably, male DALYs declined with an AAPC of −5.32%, while female DALYs decreased slightly less sharply (AAPC: -3.41%). YLD rates dropped in both sexes, but the decline was steeper in men (−4.68%) compared to women (−1.80%). YLLs, reflecting premature mortality, exhibited the sharpest reductions, especially in women (−9.01%) versus men (AAPC: −6.30%). Collectively, these results underscore sustained progress in reducing the burden of nutritional deficiencies across multiple dimensions, with men showing more rapid declines, yet women consistently experiencing higher baseline rates in several indicators.

Supplementary Tables S5–S13 present sex-specific temporal trends in the burden of different types of nutritional deficiencies in China from 1990 to 2021. Across 1990–2021, joinpoint analyses show broadly favorable trends, with important heterogeneity by deficiency and metric. Dietary iron deficiency declined steadily in prevalence, DALYs, and YLDs for both sexes, with successive negative APC segments from the mid-1990s through 2021 (e.g., prevalence AAPC ≈ −2.5 to −3.5%; 1996–2018 APC −1.9 to −3.5%). Iodine deficiency exhibited an early-2000s rise (2000–2005 APC ≈ +5% to +6%), followed by sustained declines from ~2005 onward in both incidence and prevalence. Vitamin A deficiency decreased consistently in incidence and prevalence throughout 1995–2021, with sharper male declines in several segments; however, DALY/YLD trends flattened and showed a small uptick after 2015 (APC ~ +0.27%). For “other nutritional deficiencies,” DALYs, YLDs, and YLLs generally fell across multiple joinpoints from the 1990s through the late 2010s. Protein-energy malnutrition showed the most pronounced mortality and DALY/YLL reductions—especially around 2000–2007 (e.g., DALYs/YLLs APC often −14% to −20%)—with continued though slower declines thereafter; incidence patterns were more mixed, including brief increases in the early 2010s. Collectively, these tables indicate sustained long-term improvements, punctuated by period-specific inflections differing by nutrient and metric.

### Age-period-cohort trends in incidence, prevalence, and DALYs of nutritional deficiencies in China

**Figure 5:**
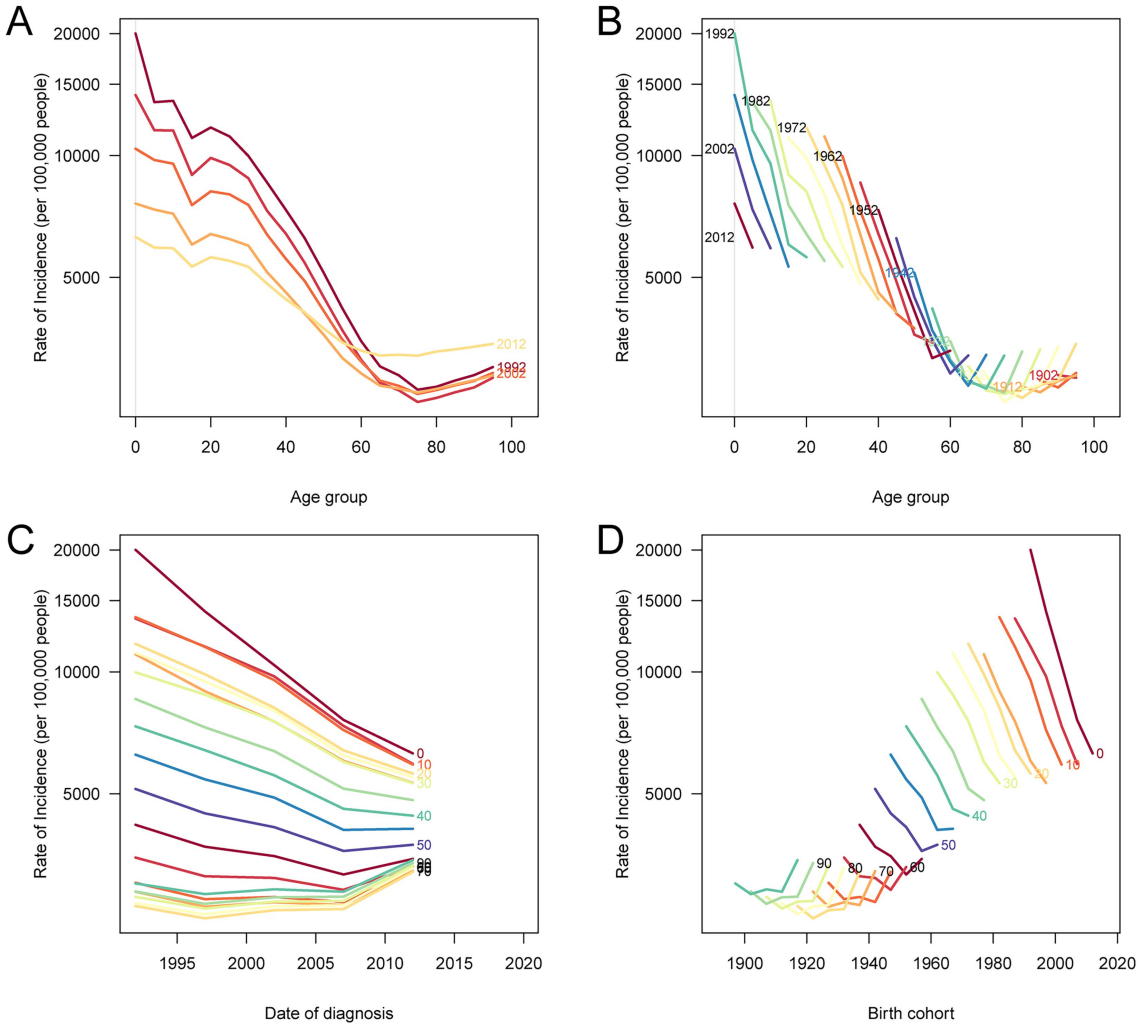
Age-period-cohort analysis of age-standardized incidence rates of nutritional deficiency in China, 1990–2021. **(A)** Age-specific incidence rates by period: each line represents a 5-year period and connects incidence rates across age groups. **(B)** Age-specific incidence rates by birth cohort: each line represents a 5-year birth cohort and connects incidence rates across age groups. **(C)** Period-specific incidence rates by age: each line represents an age group and connects incidence rates across calendar periods. **(D)** Birth cohort-specific incidence rates by age: each line represents an age group and connects incidence rates across birth cohorts.

Figure 5 and Supplementary Figures S4, S5 reveal notable age-period-cohort dynamics in the burden of nutritional deficiencies in China. In Figure 5, incidence rates decreased substantially across all age groups under 50 years from 1992 to 2012, with the most marked decline observed in children under 5 years. In contrast, individuals over 50 years experienced a decreasing trend until 2007, followed by a gradual increase thereafter. Birth cohort analysis further demonstrated that individuals born more recently had significantly lower incidence rates at the same age compared to those in earlier cohorts, reflecting sustained intergenerational improvements. Supplementary Figure S4 illustrates a continued decline in prevalence across age groups, though the reduction was modest and plateaued among older cohorts. Moreover, newer cohorts consistently showed lower prevalence across all ages. Supplementary Figure S5 highlights a sharp decline in DALYs over time for all age groups, with a slower reduction observed in the elderly (≥80 years). Together, these findings emphasize the effectiveness of nutritional policies and early-life interventions, while also highlighting the need for enhanced support for the aging population.

### Decomposition of drivers of nutritional deficiency burden in China, 1990–2021

**Figure 6:**
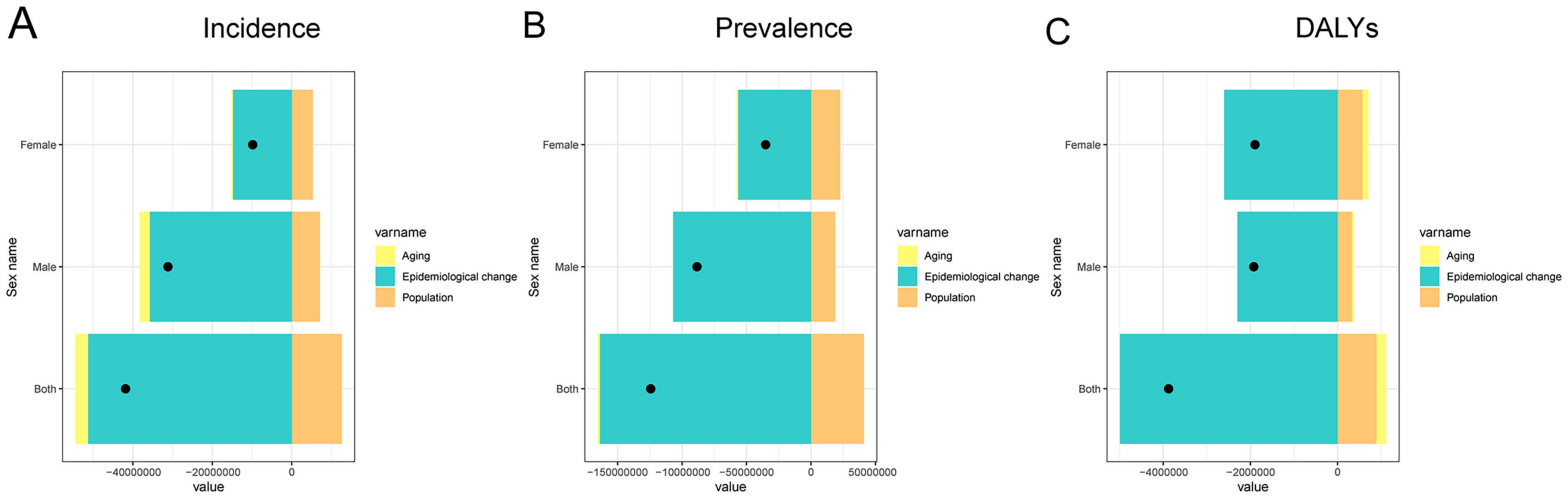
Contributions of aging, population growth, and epidemiological change to changes in the burden of nutritional deficiencies in China by sex, 1990–2021. **(A)** Decomposition of changes in incidence. **(B)** Decomposition of changes in prevalence. **(C)** Decomposition of changes in DALYs. Abbreviation: DALYs, disability-adjusted life years.

Figure 6 presents the decomposition analysis of changes in the burden of nutritional deficiencies in China from 1990 to 2021, stratified by sex. The overall changes in incidence, prevalence, and DALYs were primarily driven by a combination of population aging, population growth, and epidemiological changes. Among these, epidemiological change contributed the most significant reduction in incidence, prevalence, and DALYs across both sexes, reflecting substantial improvements in disease prevention and health interventions. However, population growth exerted a substantial upward pressure on all indicators, especially among females, due to the increasing proportion of elderly individuals. Population aging also contributed to the overall rise in disease burden, albeit to a lesser extent than aging. The overall shifts in disease burden indicate that, although significant advances have been made in lowering age-specific risks, these achievements have been partly counterbalanced by demographic changes, especially population aging. This highlights the pressing need for focused interventions to address the growing nutritional challenges associated with an aging population.

### Projected trends in nutritional deficiency burden in China by sex (2022–2030)

**Figure 7:**
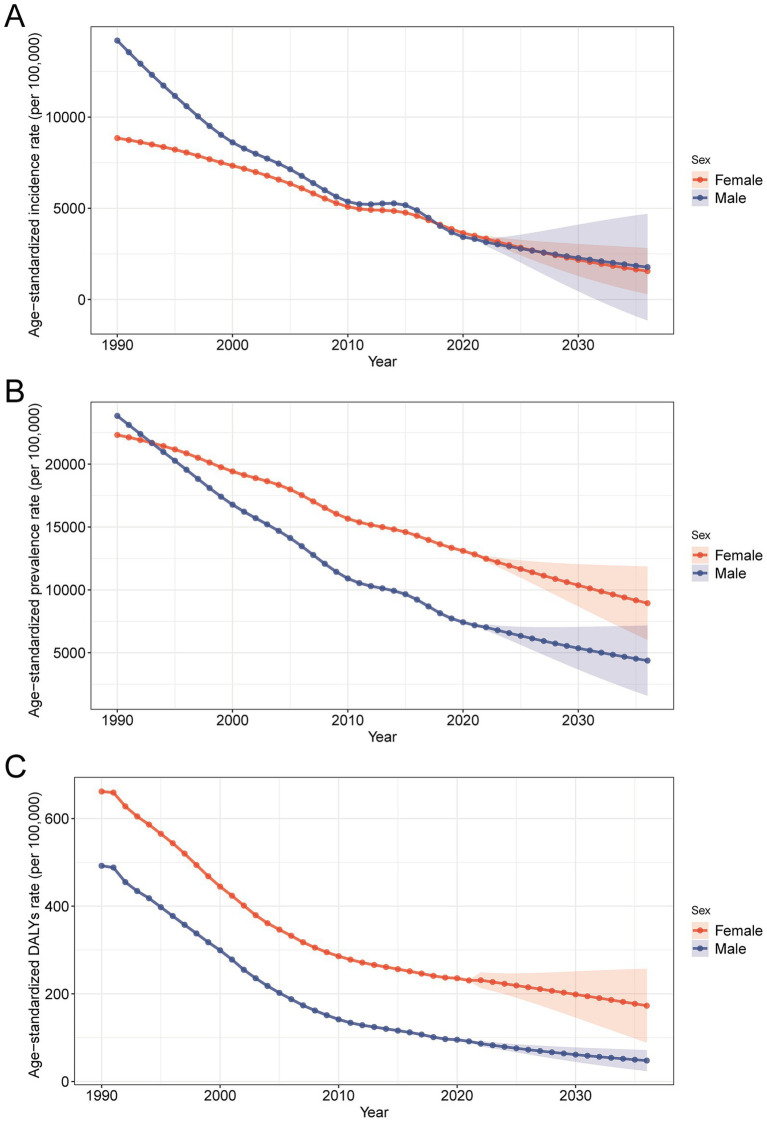
Projected trends in age-standardized rates of nutritional deficiency burden in China by sex, 2022–2030, based on Bayesian age-period-cohort analysis. **(A)** Predicted age-standardized incidence rates. **(B)** Predicted age-standardized prevalence rates. **(C)** Predicted age-standardized DALY rates. Abbreviations: DALYs, disability-adjusted life years.

Figure 7 illustrates the projected trends in age-standardized incidence, prevalence, and DALY rates for nutritional deficiencies in China from 2022 to 2030, stratified by sex. Overall, both males and females are expected to experience a continued decline in incidence and prevalence rates. The decrease in incidence is more pronounced in males, leading to a gradual convergence with the lower female incidence rate. In terms of prevalence, however, females consistently exhibit higher projected rates than males across the entire forecast period, indicating a sustained sex gap in the disease burden. Additionally, the DALY rates for both sexes are projected to decrease steadily through 2030, although females are expected to maintain higher DALY levels than males throughout the projection period. These projections highlight ongoing improvements in public health but also emphasize the need for targeted strategies to address the persistent and disproportionate burden of nutritional deficiencies among women, especially in reproductive and older age groups, where nutritional demands are typically higher.

## Discussion

This study provides a comprehensive evaluation of the long-term trends and projected burden of nutritional deficiencies in China from 1990 to 2021, using data derived from the GBD 2021. The findings revealed significant reductions across multiple burden indicators, including incidence, prevalence, mortality, DALYs, YLDs, and YLLs, for both males and females. Notably, the most substantial decreases occurred in children under five, reflecting improvements in early childhood nutrition and public health efforts. Despite the overall decline, disparities remain between sexes and age groups, with older adults continuing to bear a relatively higher burden, particularly in terms of prevalence and YLDs. The burden attributable to protein-energy malnutrition declined most prominently, whereas other forms such as iron deficiency and vitamin A deficiency persist, albeit at reduced levels. BAPC projections suggest that while the overall burden is expected to continue declining, female prevalence rates may remain consistently higher, indicating a need for sex-specific nutritional interventions. APC analysis further supports the notion of generational improvements in nutrition, with more recent birth cohorts experiencing substantially lower burden across all indicators. Collectively, these findings highlight the effectiveness of China’s nutritional improvement strategies over the past three decades and underscore the importance of sustained policy attention, particularly for vulnerable groups such as women and the elderly.

Several studies have explored the burden of nutritional deficiencies in China and globally, and our findings generally align with these observations. Previous analyses based on the GBD framework indicated that nutritional deficiency-related mortality and DALY rates have declined steadily in China over the past few decades, reflecting the country’s socioeconomic development and improved healthcare infrastructure (18, 30). For instance, the notable reductions in childhood undernutrition are consistent with prior work demonstrating the success of national maternal and child health programs, including food fortification initiatives and widespread immunization coverage (5). However, our study expands on these earlier findings by providing a more granular picture of age-, sex-, and cause-specific trends and by using BAPC modeling to project future patterns. While the global burden of iron deficiency and vitamin A deficiency remains substantial, particularly in low-income regions (2), the situation in China shows substantial improvement, although not yet eradicated. Compared to high-income countries, the residual burden among older Chinese adults, particularly women, remains relatively elevated (31, 32). This is likely due to the cumulative effects of chronic micronutrient deficiencies combined with persistent gender-specific disparities in health status and access to care. Additionally, our study confirms the downward trend in protein-energy malnutrition, as previously observed in regional and national surveys, but highlights the need for continued surveillance of micronutrient deficiencies that may not be captured by aggregate improvements (18). These comparisons underscore the value of disaggregated, long-term surveillance in guiding effective nutritional interventions.

Our age-period-cohort analysis revealed important temporal and demographic dynamics underlying the burden of nutritional deficiencies in China. The age effect demonstrated that infants and the elderly consistently bear the highest burden, reflecting the greater vulnerability of these age groups due to immature or declining physiological function and immunity (33, 34). Period effects showed a general decline across all age groups since the early 2000s, coinciding with significant national health initiatives such as the expansion of basic public health services and nutritional supplementation programs (35). However, the cohort effects suggested notable intergenerational improvements, as more recent birth cohorts experienced substantially lower incidence and DALYs across all ages. This trend likely reflects improved prenatal care, early childhood nutrition, and overall living standards. Importantly, sex disparities were evident throughout the study period. Women, particularly older women, consistently exhibited higher prevalence, YLDs, and DALY rates than men, a pattern likely influenced by a combination of biological and socio-cultural determinants. Physiologically, women are more vulnerable to micronutrient deficiencies due to factors such as iron loss from menstruation, increased nutritional demands during pregnancy and lactation, and longer life expectancy, which prolongs exposure to nutritional stressors over the life course (36–38). Moreover, the apparent sex gap in prevalence and DALYs among individuals aged 20–49 may be partially attributed to sex differences in healthcare-seeking behavior and reporting practices. For instance, micronutrient deficiencies may be more frequently identified in women due to routine screening during antenatal care, whereas such deficiencies might remain underreported in men, who typically have fewer contacts with preventive health services during early and mid-adulthood. However, beyond biology, socio-cultural dynamics in China may exacerbate these disparities. Traditional intra-household food allocation practices often prioritize men and children, potentially reducing women’s access to nutrient-rich foods, especially in rural or resource-limited settings. Additionally, women may have limited access to healthcare or delay seeking treatment due to caregiving responsibilities, financial dependence, or gendered health-seeking behaviors. Economic inequities, lower educational attainment, and occupational exposures also play roles in compromising women’s nutritional status.

Another biological factor contributing to sex differences in nutritional outcomes, particularly in the context of acute malnutrition, is immune function. Females generally mount stronger immune responses than males, influenced by hormonal and genetic factors, which may provide greater protection against infections (37). In contrast, men are more susceptible to infectious diseases, which can worsen nutritional status through the infection-malnutrition cycle—a bidirectional process where malnutrition impairs immunity and increases infection risk, which in turn exacerbates malnutrition (39). In contrast, higher mortality and YLLs observed in men, particularly in earlier years, may reflect greater exposure to acute deprivation, risk-taking behavior, or reluctance to engage with preventive care. Biological contributors such as differences in hormonal regulation, immune function, and metabolic rates may further influence these divergent patterns (40, 41). Collectively, these findings highlight the importance of integrating sex-specific and gender-sensitive strategies in national nutrition programs, targeting both physiological needs and structural inequalities to ensure more equitable health outcomes.

The observed decline in the burden of nutritional deficiencies in China over the past three decades highlights the effectiveness of several national public health interventions. As shown in the Joinpoint regression analysis, periods of accelerated decline in age-standardized prevalence and DALYs rates, particularly after the early 2000s, align with major health system reforms and the implementation of national nutrition programs. Programs such as the such as “Outline of the Healthy China 2030 Plan” and “Healthy China Action Plan (2019–2030),” and maternal and child health initiatives have contributed to improving dietary diversity, fortifying staple foods, and providing targeted supplementation to high-risk groups, particularly infants, pregnant women, and the elderly (42). This inflection points identified in our analysis suggest that such policy shifts had a measurable impact on burden reduction, reinforcing the value of sustained national investment in nutrition and health infrastructure. Nevertheless, the remaining burden, especially among older adults and women, underscores the need to strengthen health policies that address life-course nutrition. Clinically, early screening for malnutrition in both primary care and hospital settings should be expanded, particularly for vulnerable populations such as the elderly and those with chronic illnesses (14, 43–45). Incorporating regular nutritional evaluations into primary and geriatric healthcare settings could significantly enhance the early identification and management of malnutrition, leading to better clinical outcomes and a reduction in overall healthcare expenditures (46). On the policy front, gender-sensitive strategies should be adopted to account for sex-based biological and socioeconomic differences. For example, ensuring equitable access to iron and folic acid supplementation and promoting nutrition education among women of reproductive age are essential for long-term gains (47). Additionally, given the generational improvements indicated by cohort analysis, sustained investment in early childhood nutrition remains essential. Initiatives such as the promotion of breastfeeding, targeted micronutrient supplementation, and the expansion of school-based meal programs are critical for supporting long-term health and development (48–51). These actions, aligned with the Healthy China 2030 blueprint, are critical for sustaining progress and ensuring that nutritional equity is achieved across all demographic groups.

Despite providing a comprehensive assessment of nutritional deficiency trends in China, this study has several limitations. First, our analysis relies entirely on estimates from the GBD 2021 study, which synthesizes data from a wide range of sources and uses complex statistical models to address data gaps. While this approach enhances coverage and comparability, it introduces uncertainties due to possible underreporting, misclassification, or geographic imbalance in the underlying data. These limitations are particularly relevant for earlier years and less commonly tracked micronutrients (20, 52). The 95% UIs presented—derived from 1,000 posterior draws—reflect variability from data inputs and modeling assumptions. Wider UIs, especially in projections and smaller subgroups, suggest limited precision, and overlapping intervals indicate that differences or trends may not be statistically significant. Projections, therefore, should be interpreted as directional estimates rather than precise forecasts. Second, several micronutrient deficiencies—such as vitamin D, selenium, and folate—are not disaggregated in the GBD framework due to insufficient primary data and are instead grouped under “other nutritional deficiencies” (53). This aggregation limits the ability to discern their individual contributions to disease burden and represents a key knowledge gap. Future efforts should prioritize expanding nutritional surveillance systems and enhancing the granularity of micronutrient-specific data collection. Third, the ecological nature of our design limits causal inference, and we were unable to incorporate individual-level variables such as income, education, or dietary intake patterns. Future studies integrating cohort designs, machine learning models, and spatial analysis techniques could better identify high-risk populations and inform targeted interventions. Enhanced nutritional monitoring, especially for vulnerable groups like young children and the elderly, will be vital for achieving China’s public health targets, including those outlined in the Healthy China 2030 initiative.

## Conclusion

This study provides a comprehensive and systematic assessment of the burden and long-term trends of nutritional deficiencies in China over the past three decades. The analysis reflects evolving demographic, epidemiological, and policy-related dynamics that shape the national nutritional health landscape. The observed patterns underscore the ongoing need for strengthened surveillance, targeted interventions, and health education strategies tailored to vulnerable populations, including children, women, and older adults. Moving forward, integrated nutrition policies that address both macro- and micronutrient deficiencies should be prioritized to achieve sustainable improvements in public health. Future studies should leverage high-quality longitudinal data, incorporate behavioral and socioeconomic risk factors, and explore the effects of environmental and dietary transitions on nutritional outcomes. Emphasizing the intersection of nutrition with chronic disease prevention, digital health tools, and equity-based approaches will be crucial for informing the next generation of national and global nutrition strategies.

## Data Availability

Publicly available datasets were analyzed in this study. This data can be found at: http://ghdx.healthdata.org/gbd-results-tool.

## Footnotes

^1^ http://ghdx.healthdata.org/gbd-results-tool

## Supplementary material

SUPPLEMENTARY FIGURE S1 Age- and sex-specific number and age-standardized rates of DALYs, YLDs, and YLLs for nutritional deficiencies in China, 2021. **(A)** Number of DALYs by age and sex. **(B)** Age-standardized DALY rate per 100,000 population by age and sex. **(C)** Number of YLDs by age and sex. **(D)** Age-standardized YLD rate per 100,000 population by age and sex. **(E)** Number of YLLs by age and sex. **(F)** Age-standardized YLL rate per 100,000 population by age and sex. DALYs, disability-adjusted life years; YLDs, years lived with disability; YLLs, years of life lost.

SUPPLEMENTARY FIGURE S2 Age-specific number and crude rates of incidence, prevalence, mortality, DALYs, YLDs, and YLLs for nutritional deficiencies in China, 1990 and 2021. **(A)** Number and crude rate of incidence per 100,000 population by age group in 1990 and 2021. **(B)** Number and crude rate of prevalence per 100,000 population by age group in 1990 and 2021. **(C)** Number and crude rate of deaths per 100,000 population by age group in 1990 and 2021. **(D)** Number and crude rate of DALYs per 100,000 population by age group in 1990 and 2021. **(E)** Number and crude rate of YLDs per 100,000 population by age group in 1990 and 2021. **(F)** Number and crude rate of YLLs per 100,000 population by age group in 1990 and 2021. DALYs, disability-adjusted life years; YLDs, years lived with disability; YLLs, years of life lost.

SUPPLEMENTARY FIGURE S3 Trends in age-standardized rates of disease burden attributable to nutritional deficiencies in China and globally, 1990–2021. **(A)** Age-standardized incidence, prevalence, mortality, DALY, YLD, and YLL rates per 100,000 population in China from 1990 to 2021. **(B)** Age-standardized incidence, prevalence, mortality, DALY, YLD, and YLL rates per 100,000 population globally from 1990 to 2021. DALYs, disability-adjusted life years; YLDs, years lived with disability; YLLs, years of life lost.

SUPPLEMENTARY FIGURE S4 Age-period-cohort analysis of age-standardized prevalence rates of nutritional deficiency in China. **(A)** Age-specific prevalence rates across successive time periods (1990–2021), with each line connecting the prevalence rate of a specific age group in each period. **(B)** Age-specific prevalence rates across successive birth cohorts, with each line connecting the prevalence rate of individuals born in a specific cohort across age groups. **(C)** Period-specific prevalence rates across age groups, with each line representing the prevalence rate of a fixed age group over different time periods. **(D)** Cohort-specific prevalence rates across age groups, with each line depicting the prevalence rate of a fixed birth cohort across age groups.

SUPPLEMENTARY FIGURE S5 Age-period-cohort analysis of DALYs due to nutritional deficiencies in China, 1990–2021. **(A)** Age-specific DALY rates across different time periods, where each line represents a 5-year period and illustrates changes in DALY rates across age groups. **(B)** Age-specific DALY rates across different birth cohorts, with each line indicating a 5-year birth cohort and showing variations in DALY rates by age within that cohort. **(C)** Period-specific DALY rates across age groups, where each line represents a 5-year age group and displays changes in DALY rates over successive time periods. **(D)** Birth cohort-specific DALY rates across age groups, with each line representing a 5-year age group and showing changes in DALY rates across different birth cohorts. DALYs, disability-adjusted life years.
