# Supplementary figures and images for: Burden, trends, and projections of nutritional deficiencies in China from 1990 to 2030
Source: Front Nutr. 2025 Sep 4;12:1643869. doi: 10.3389/fnut.2025.1643869 (PMC12444020; doi:10.3389/fnut.2025.1643869)

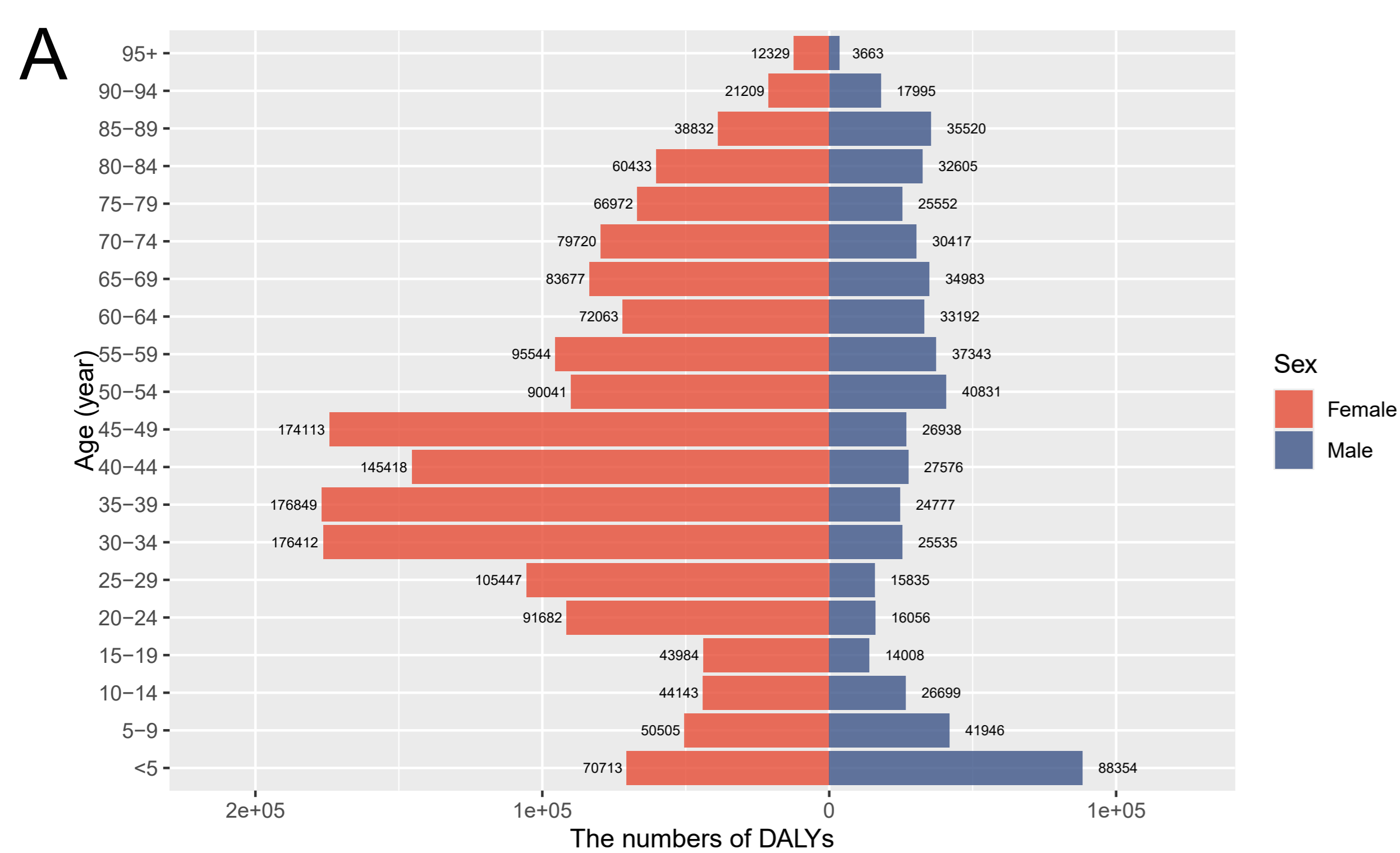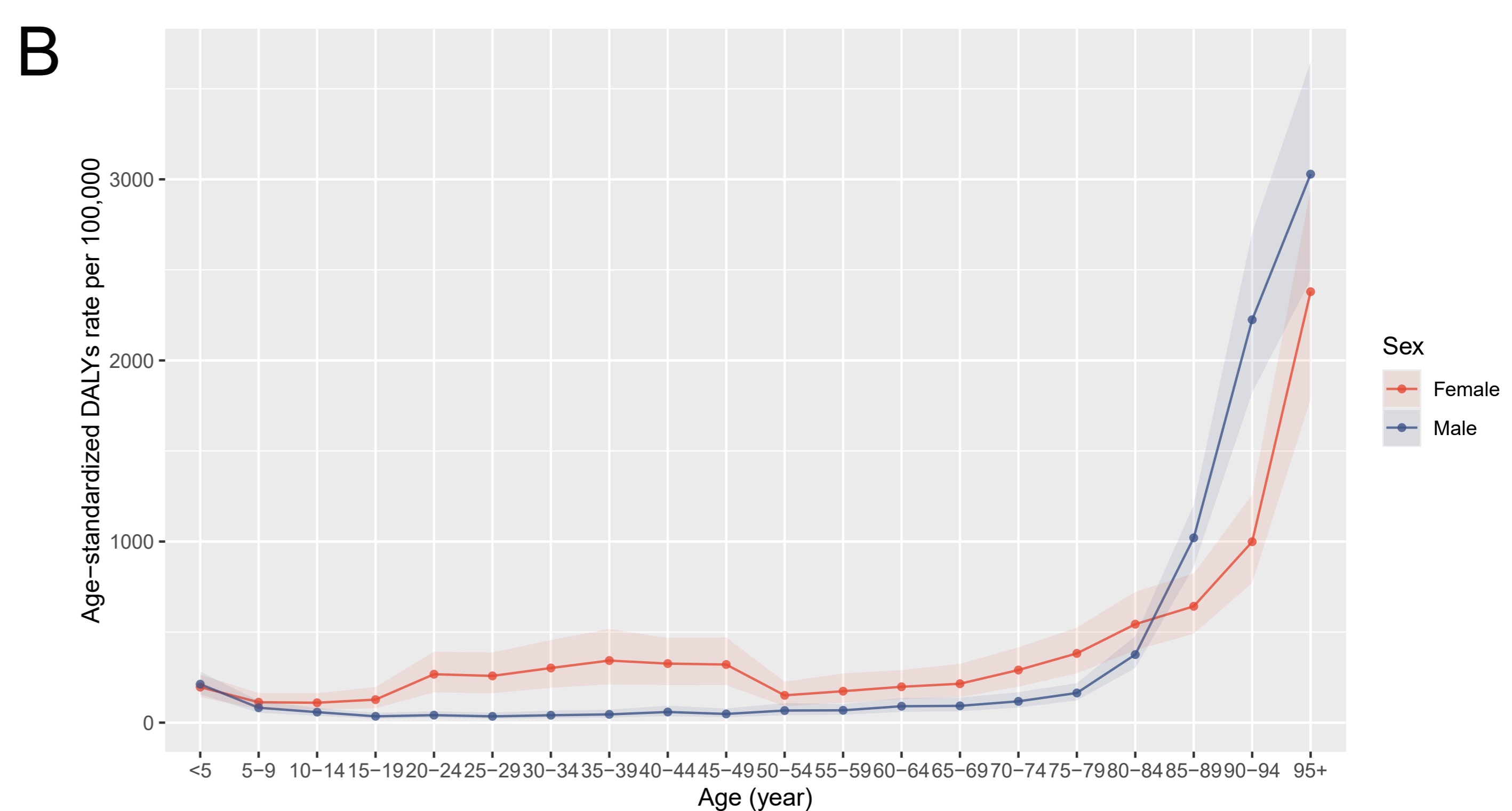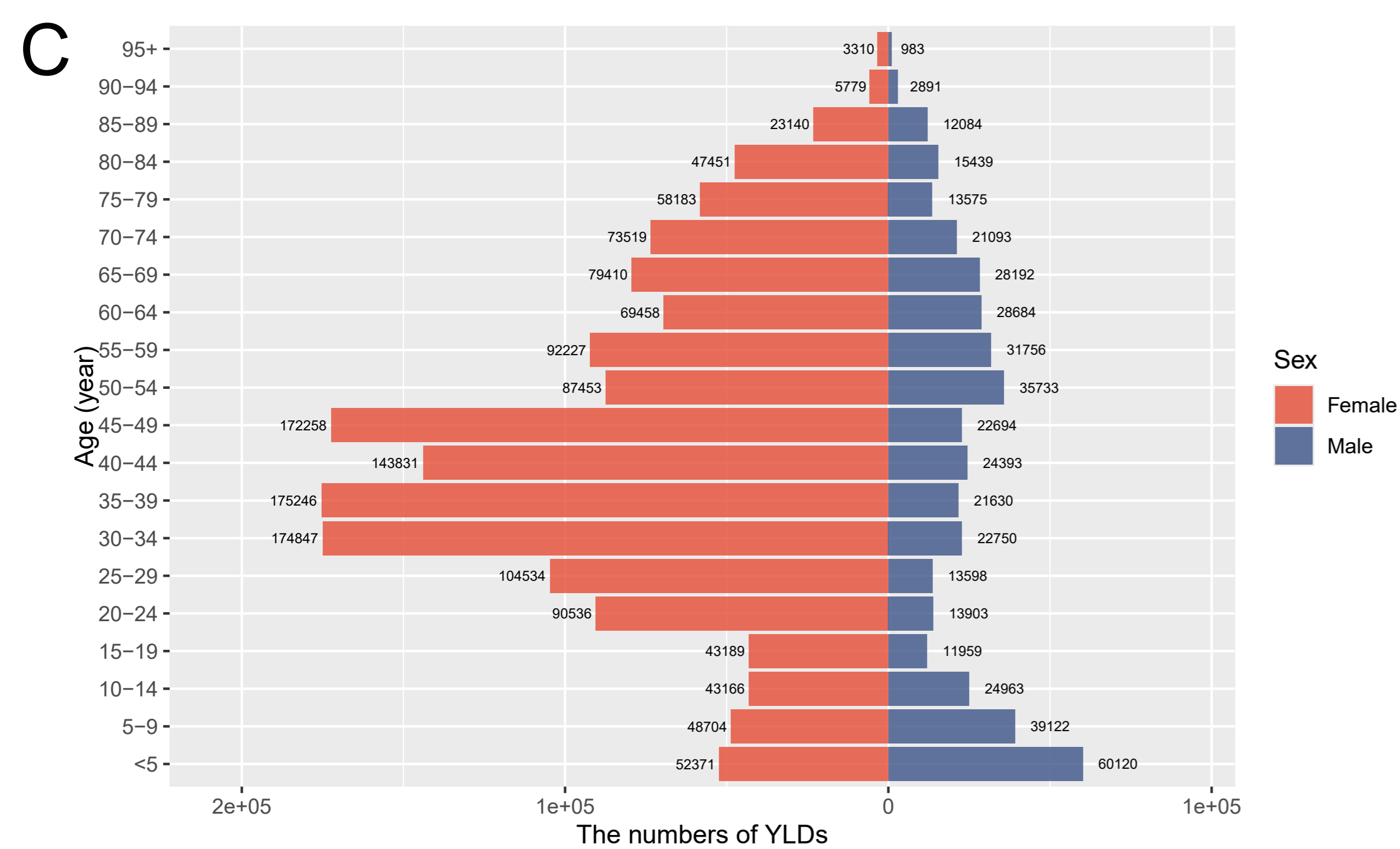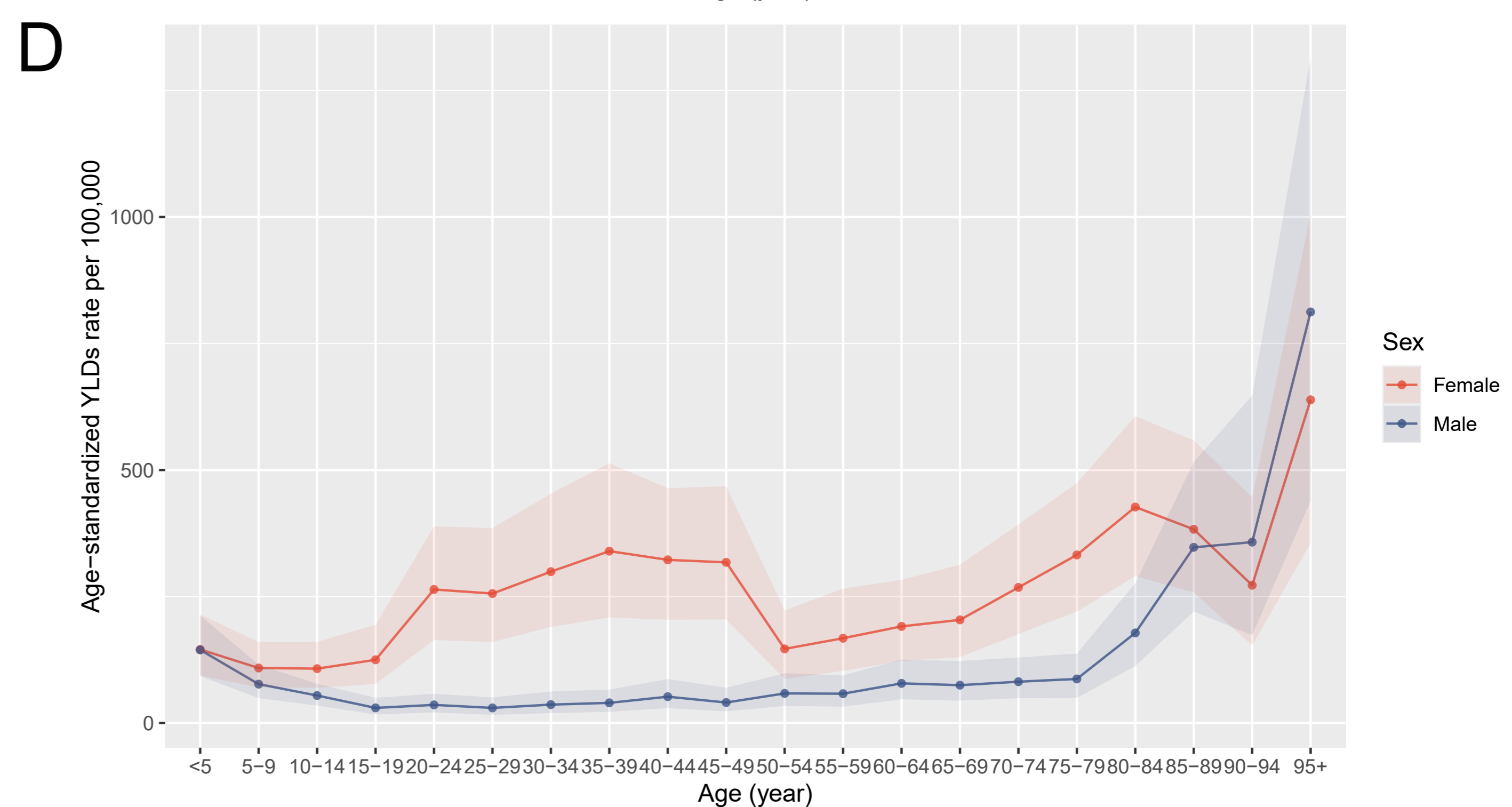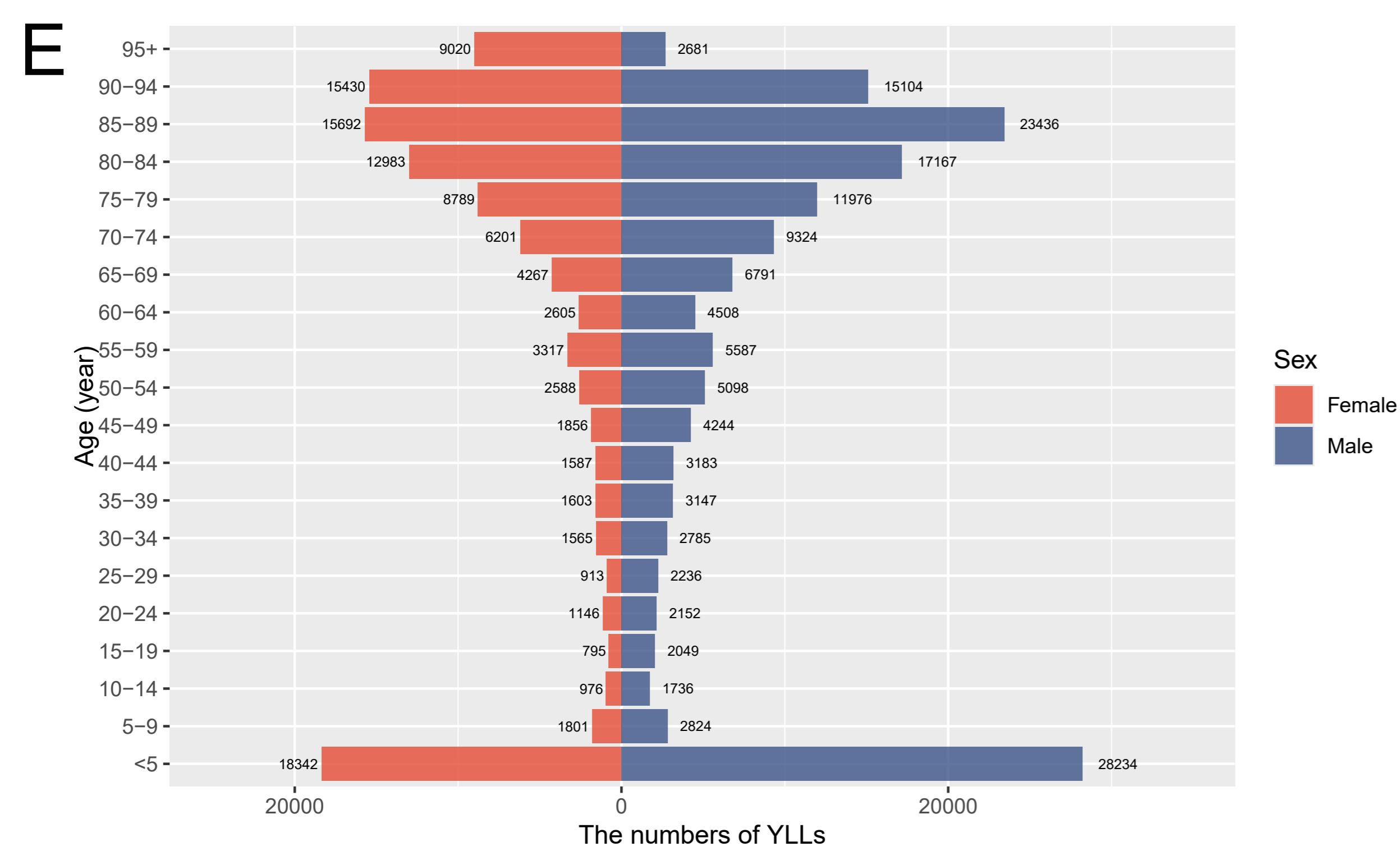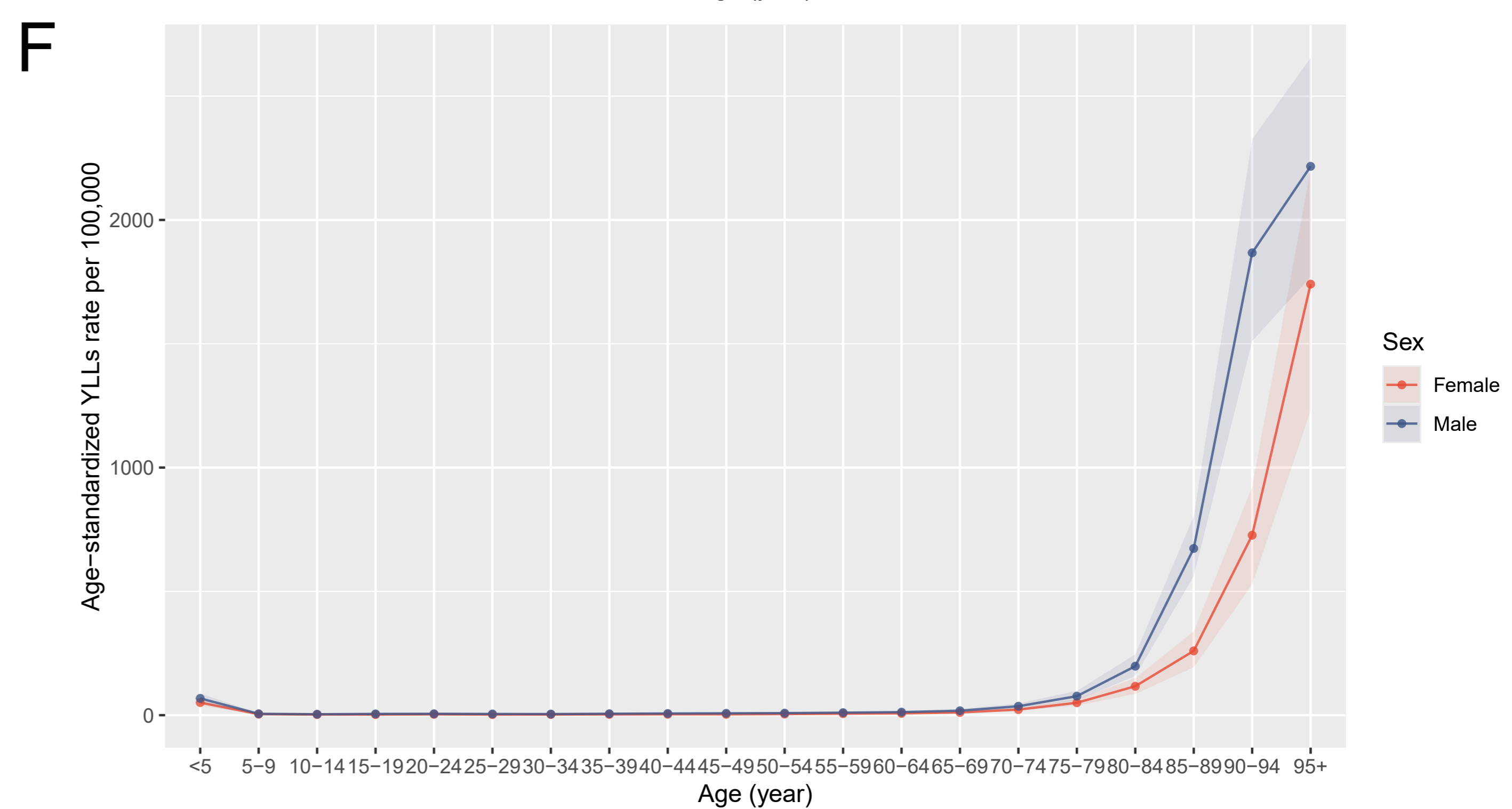

Supplement: SUPPLEMENTARY FIGURE S1 — Age- and sex-specific number and age-standardized rates of DALYs, YLDs, and YLLs for nutritional deficiencies in China, 2021. (A) Number of DALYs by age and sex. (B) Age-standardized DALY rate per 100,000 population by age and sex. (C) Number of YLDs by age and sex. (D) Age-standardized YLD rate per 100,000 population by age and sex. (E) Number of YLLs by age and sex. (F) Age-standardized YLL rate per 100,000 population by age and sex. DALYs, disability-adjusted life years; YLDs, years lived with disability; YLLs, years of life lost. [file Data_Sheet_1.PDF]

A

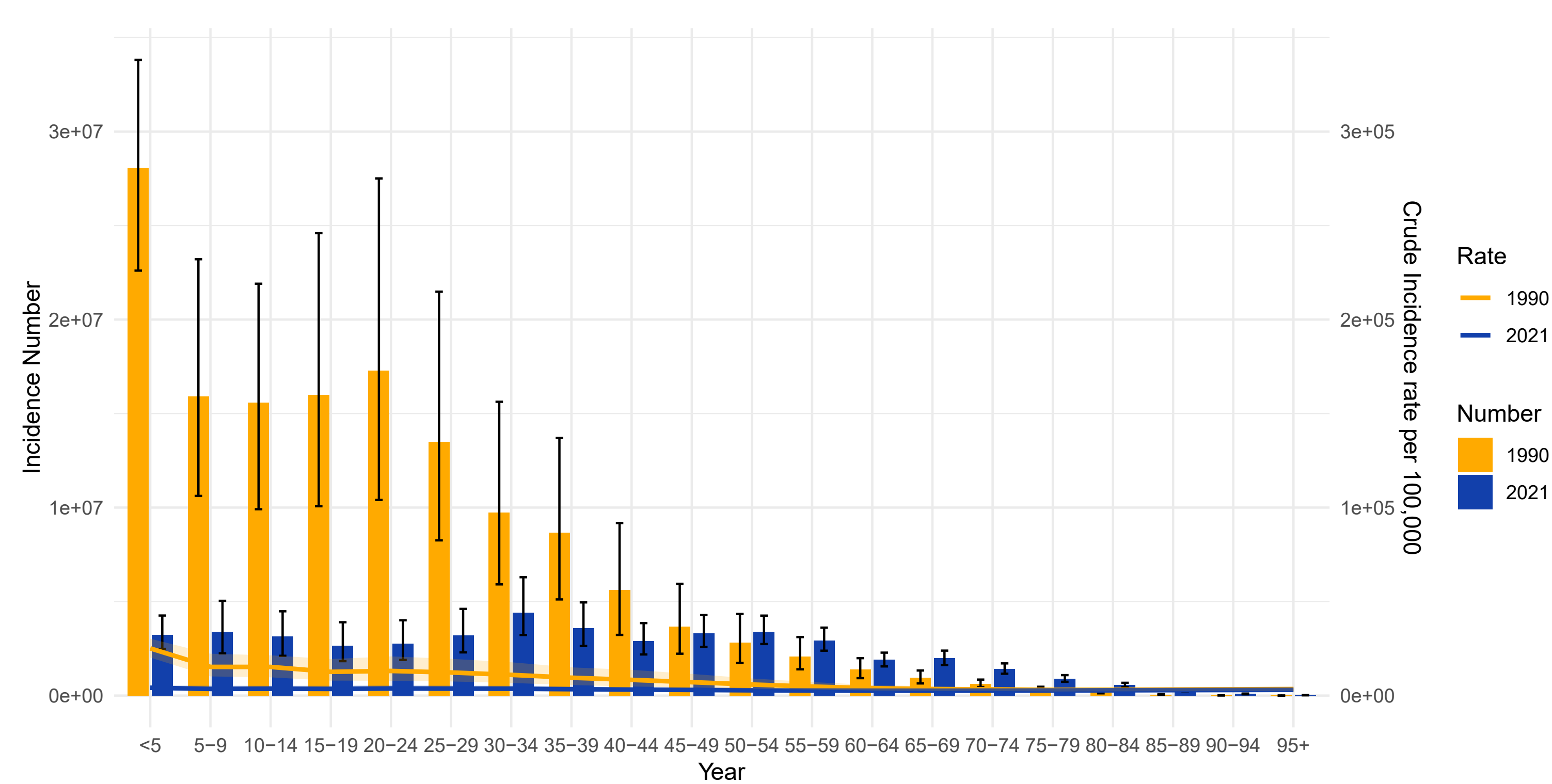

B

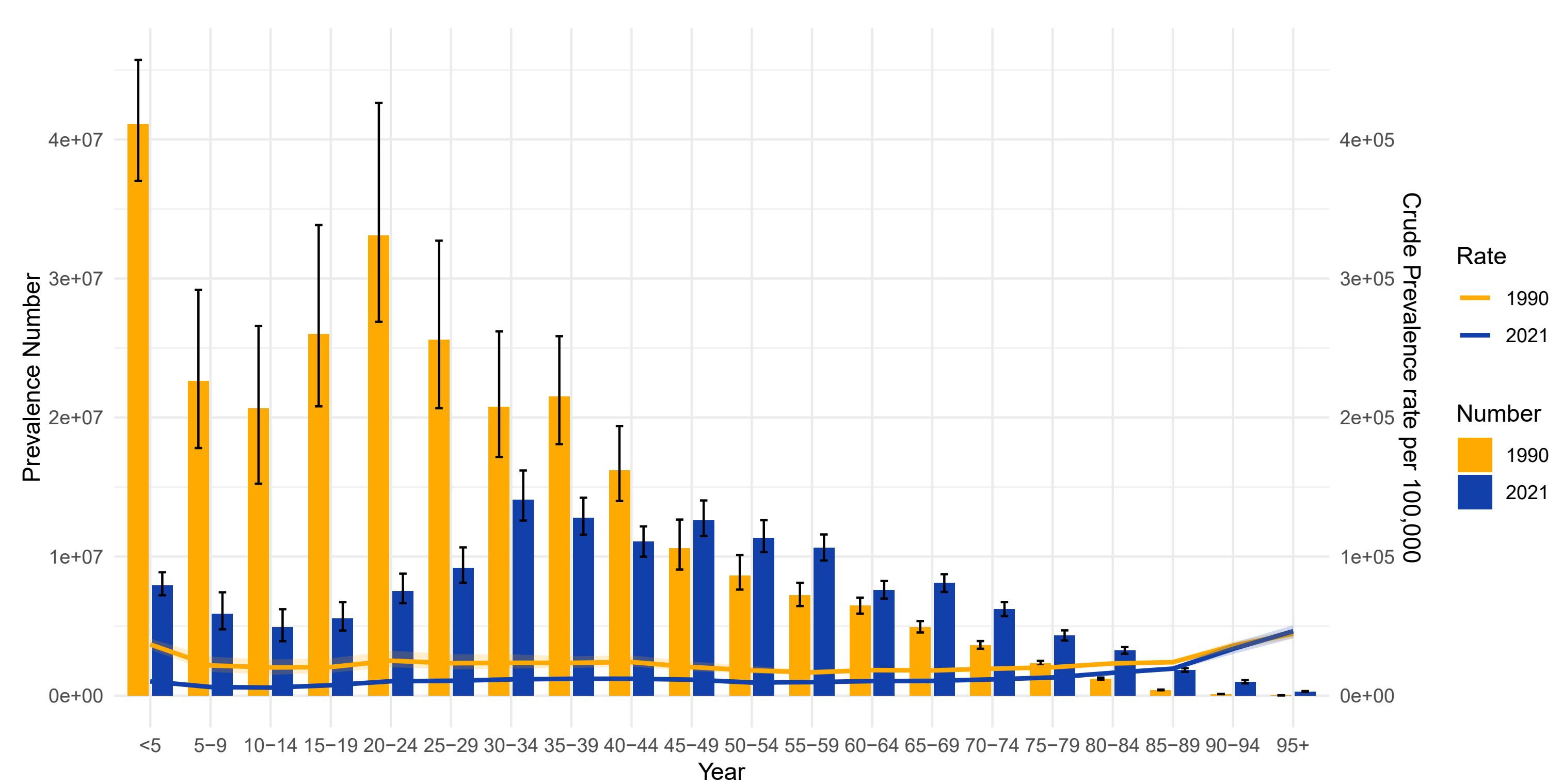

C

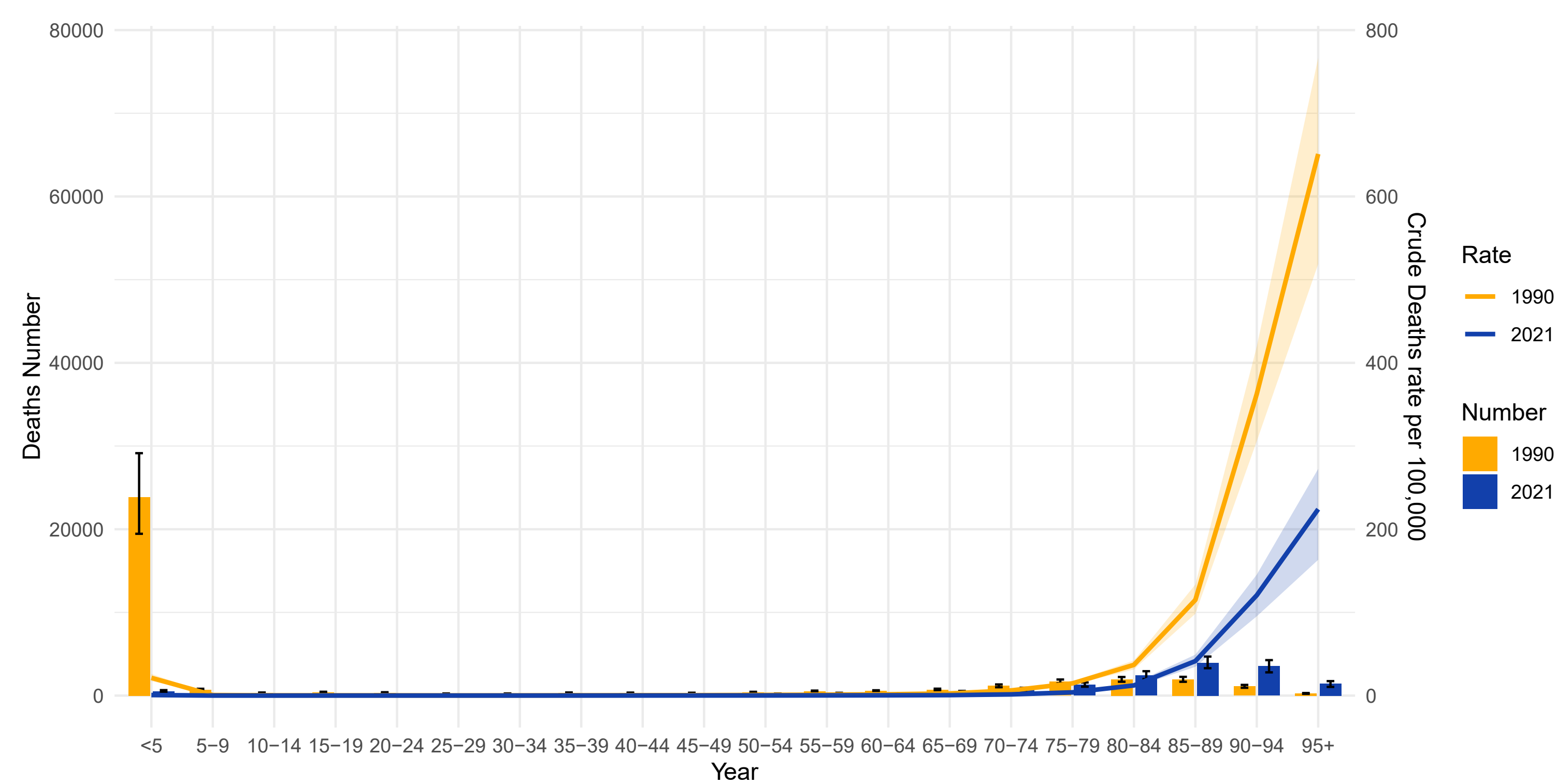

D

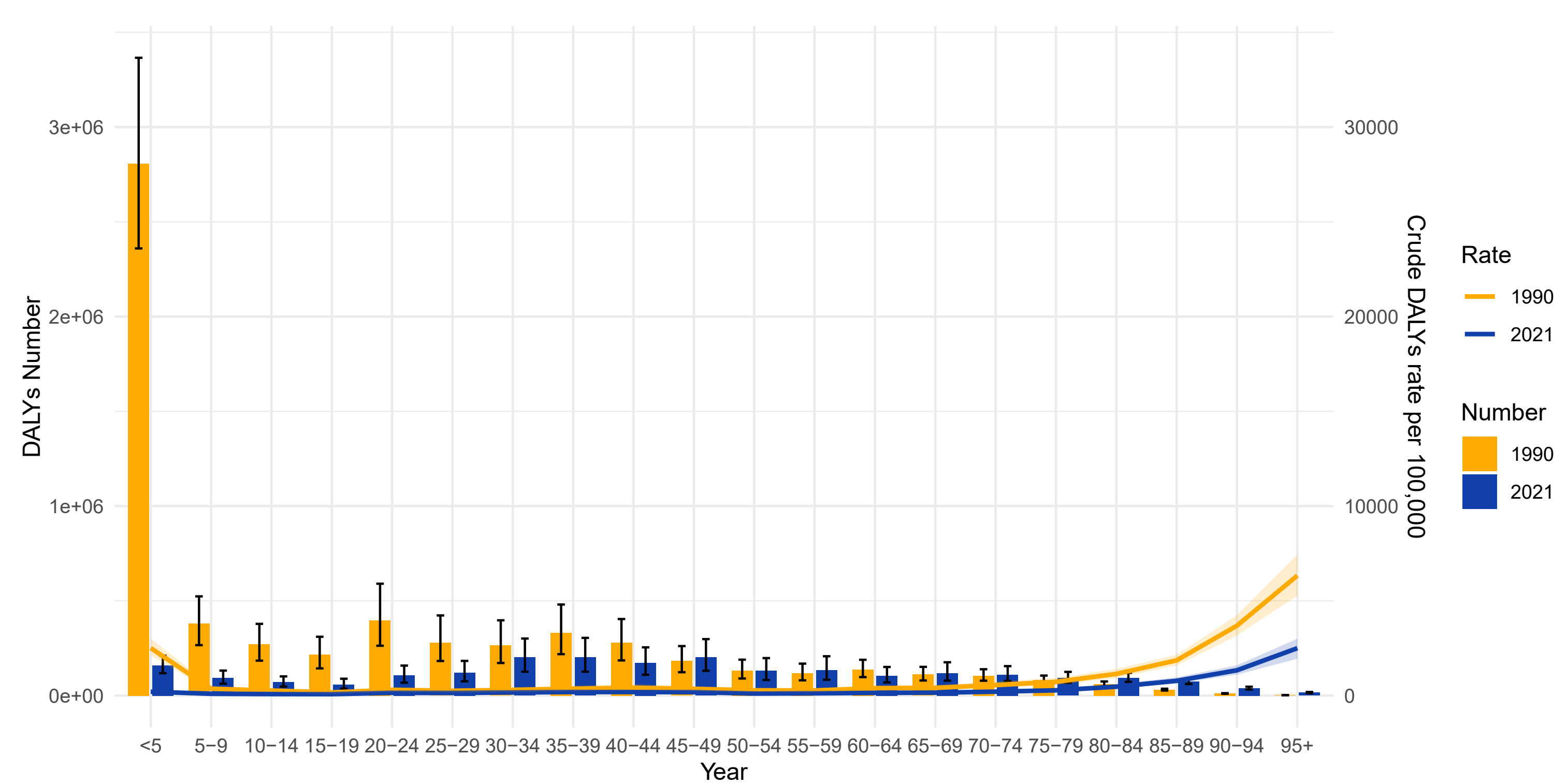

E

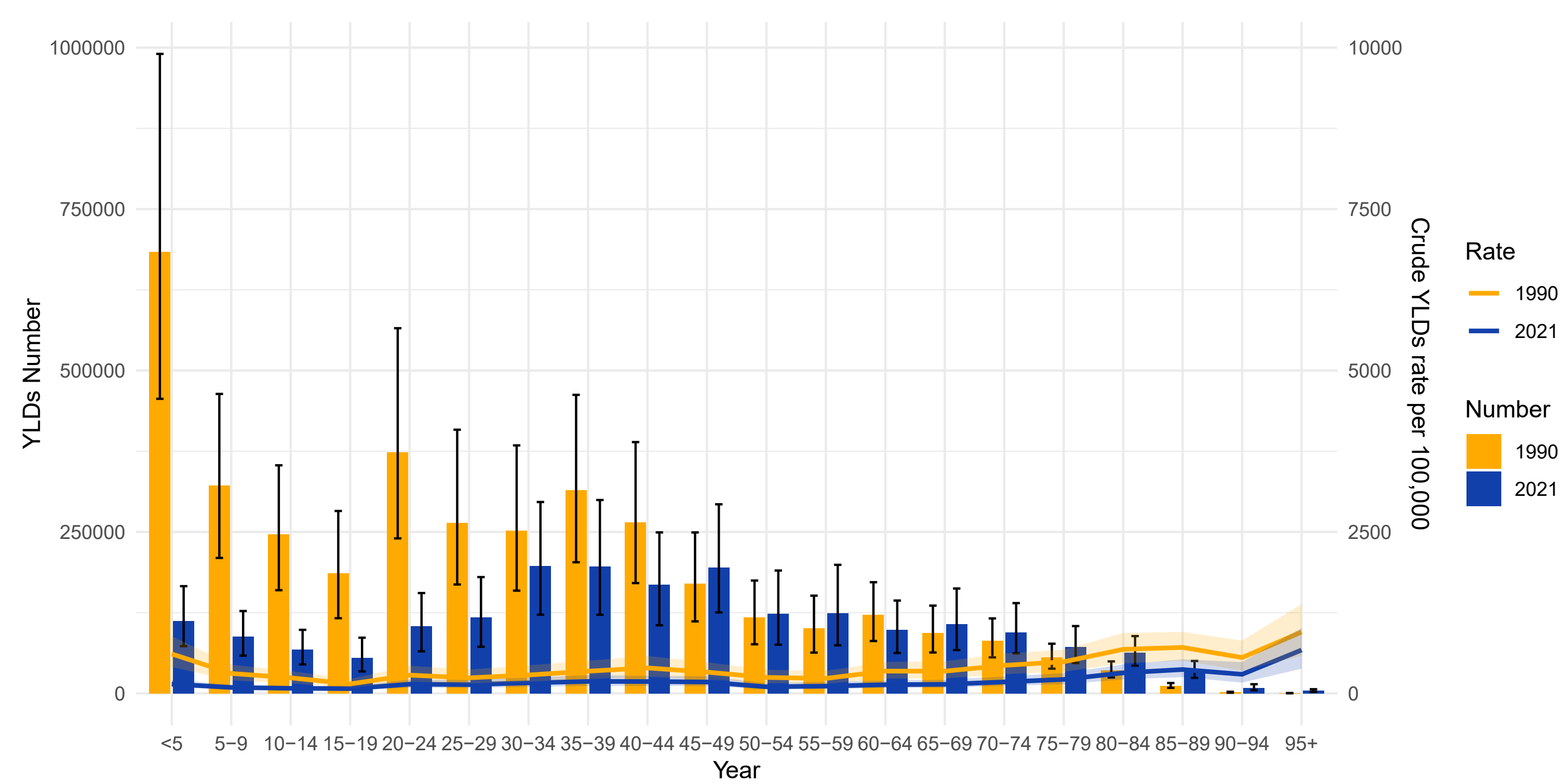

F

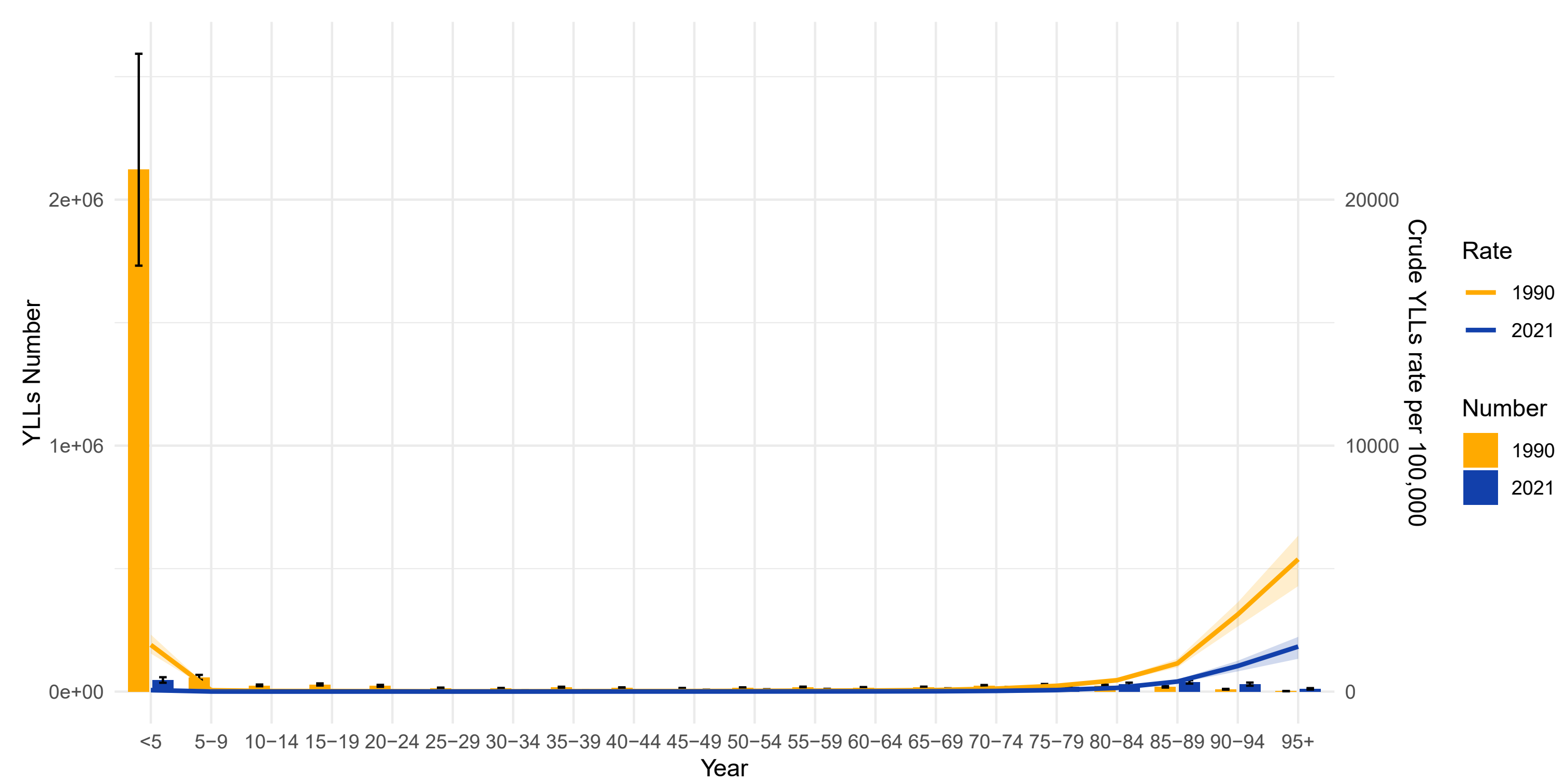

Supplement: SUPPLEMENTARY FIGURE S2 — Age-specific number and crude rates of incidence, prevalence, mortality, DALYs, YLDs, and YLLs for nutritional deficiencies in China, 1990 and 2021. (A) Number and crude rate of incidence per 100,000 population by age group in 1990 and 2021. (B) Number and crude rate of prevalence per 100,000 population by age group in 1990 and 2021. (C) Number and crude rate of deaths per 100,000 population by age group in 1990 and 2021. (D) Number and crude rate of DALYs per 100,000 population by age group in 1990 and 2021. (E) Number and crude rate of YLDs per 100,000 population by age group in 1990 and 2021. (F) Number and crude rate of YLLs per 100,000 population by age group in 1990 and 2021. DALYs, disability-adjusted life years; YLDs, years lived with disability; YLLs, years of life lost. [file Data_Sheet_2.PDF]

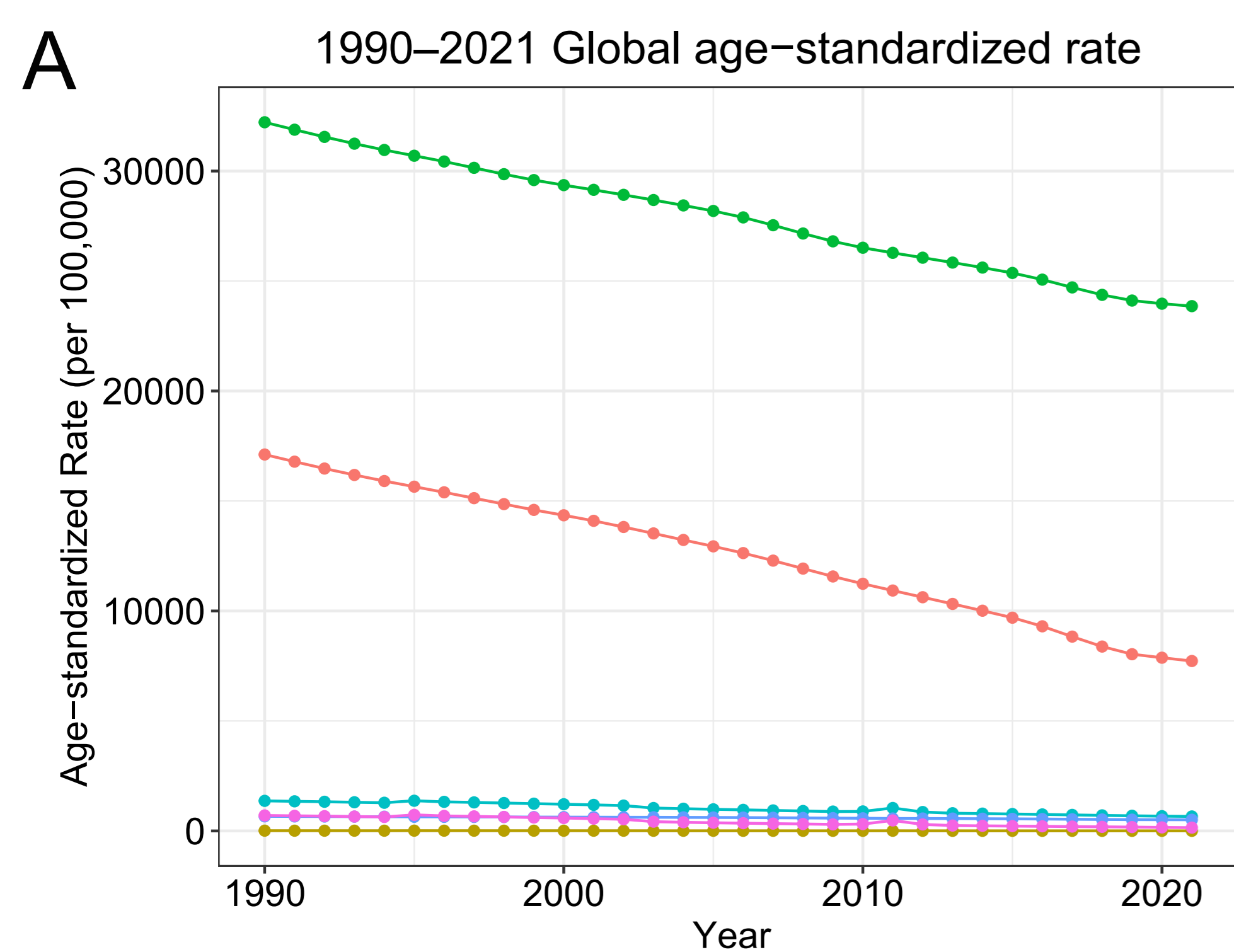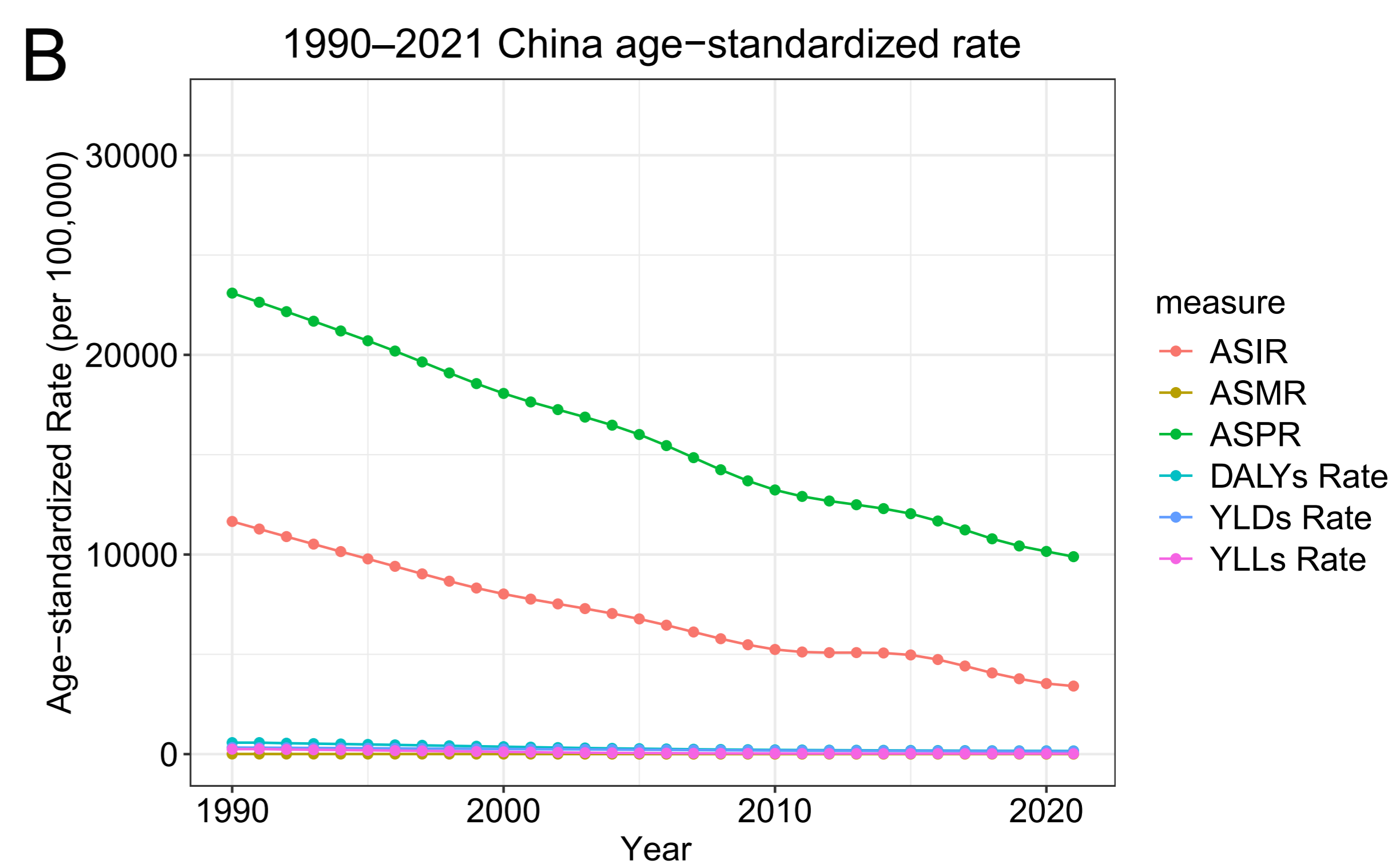

Supplement: SUPPLEMENTARY FIGURE S3 — Trends in age-standardized rates of disease burden attributable to nutritional deficiencies in China and globally, 1990–2021. (A) Age-standardized incidence, prevalence, mortality, DALY, YLD, and YLL rates per 100,000 population in China from 1990 to 2021. (B) Age-standardized incidence, prevalence, mortality, DALY, YLD, and YLL rates per 100,000 population globally from 1990 to 2021. DALYs, disability-adjusted life years; YLDs, years lived with disability; YLLs, years of life lost. [file Data_Sheet_3.PDF]

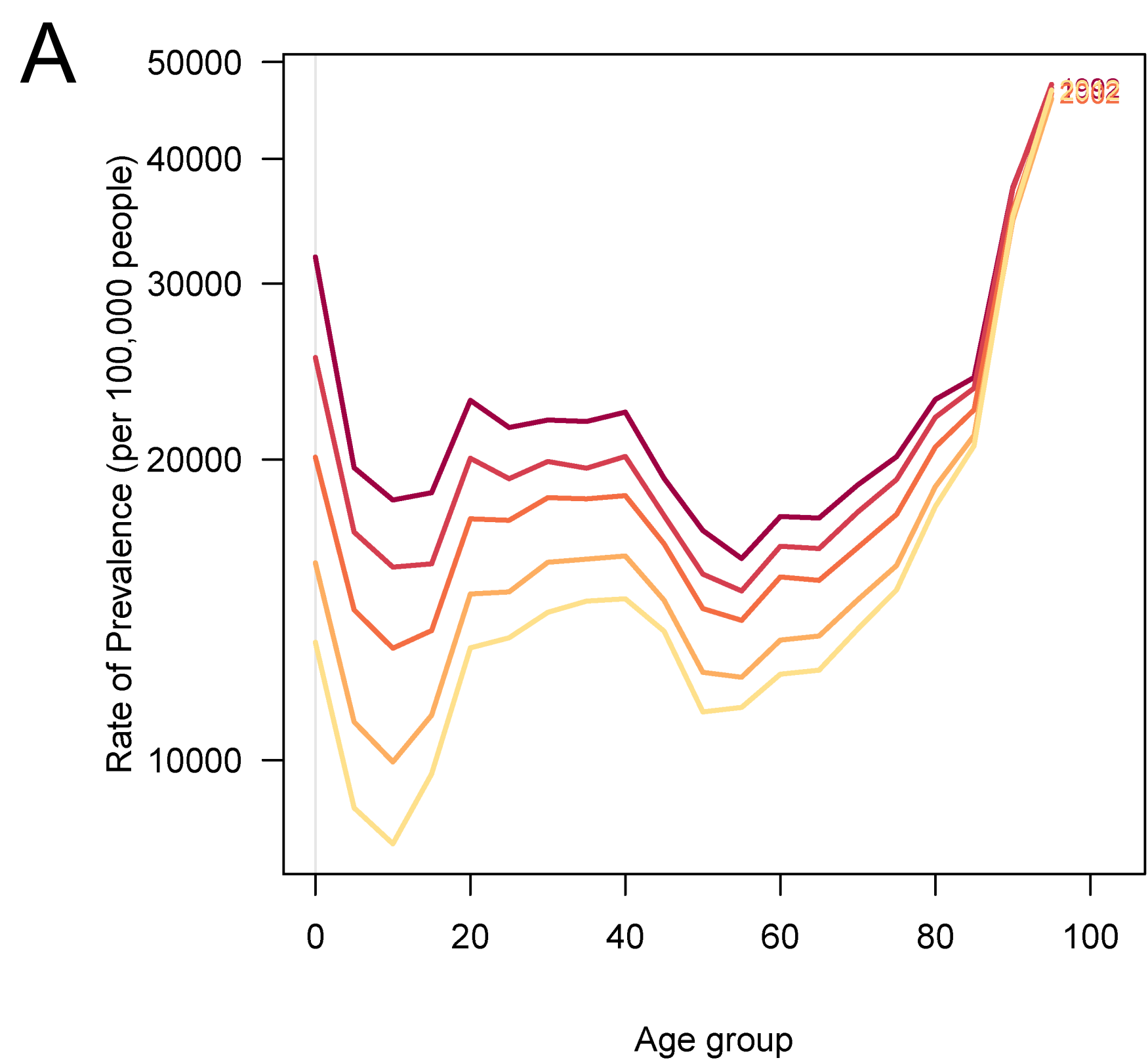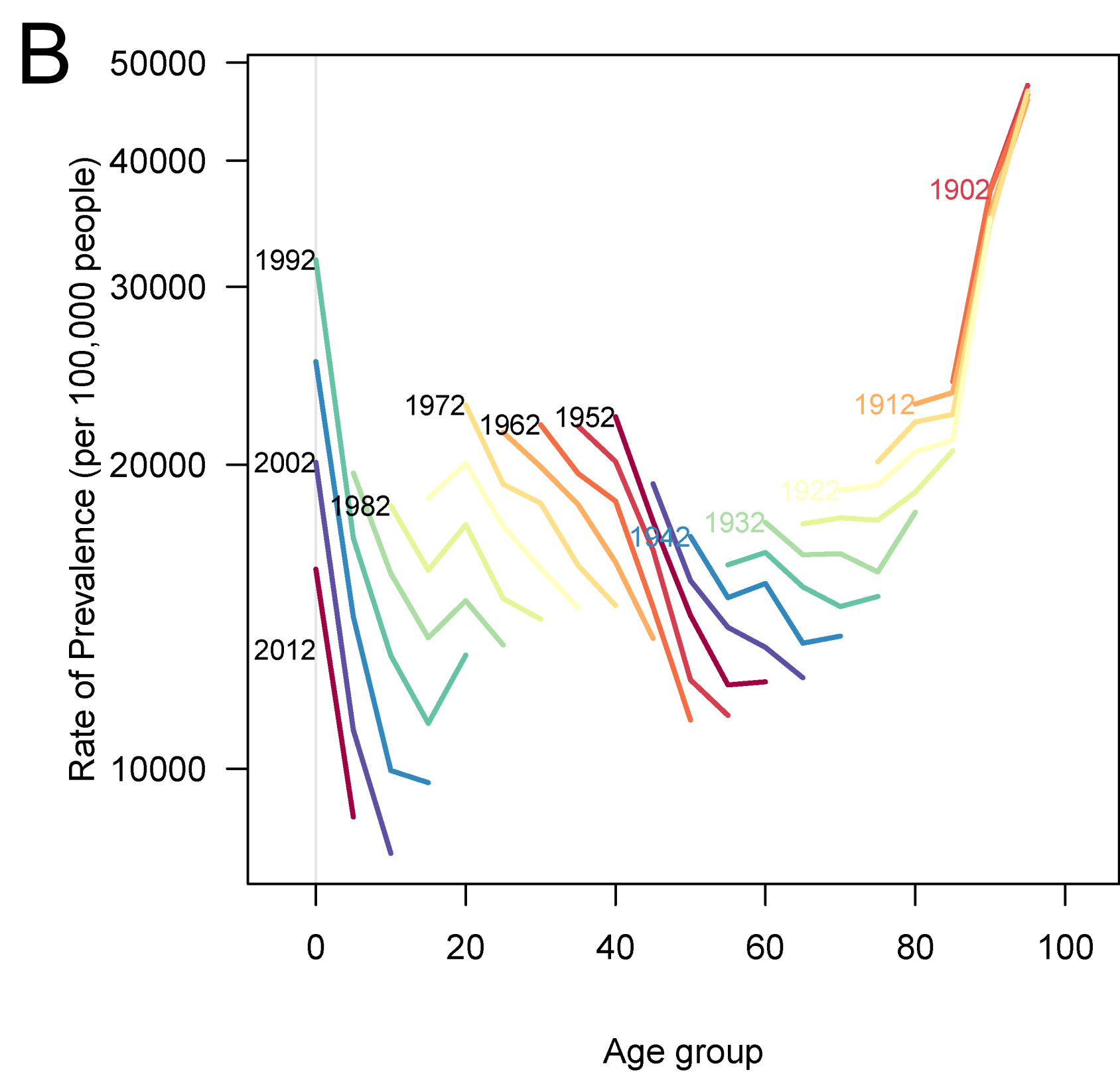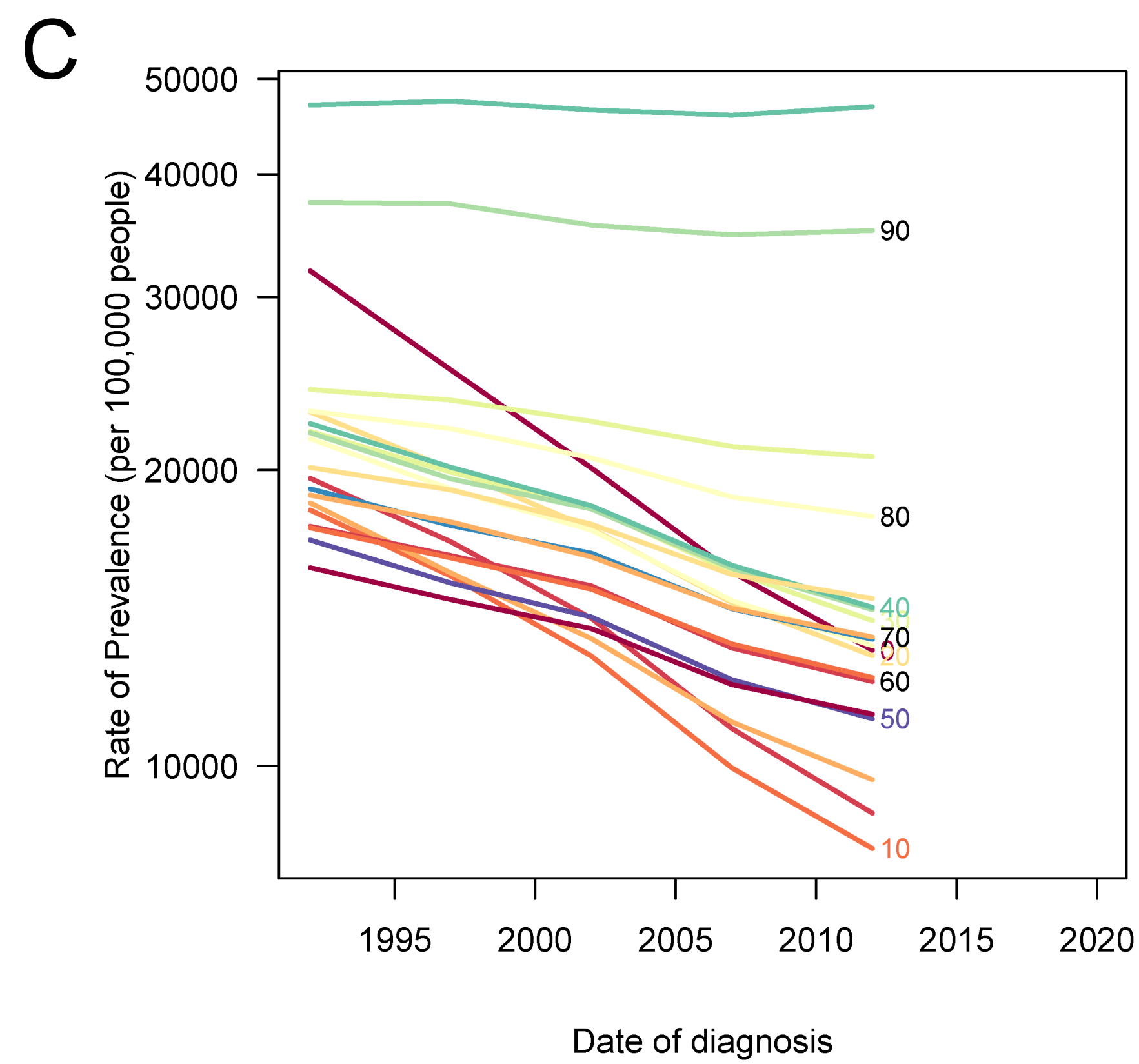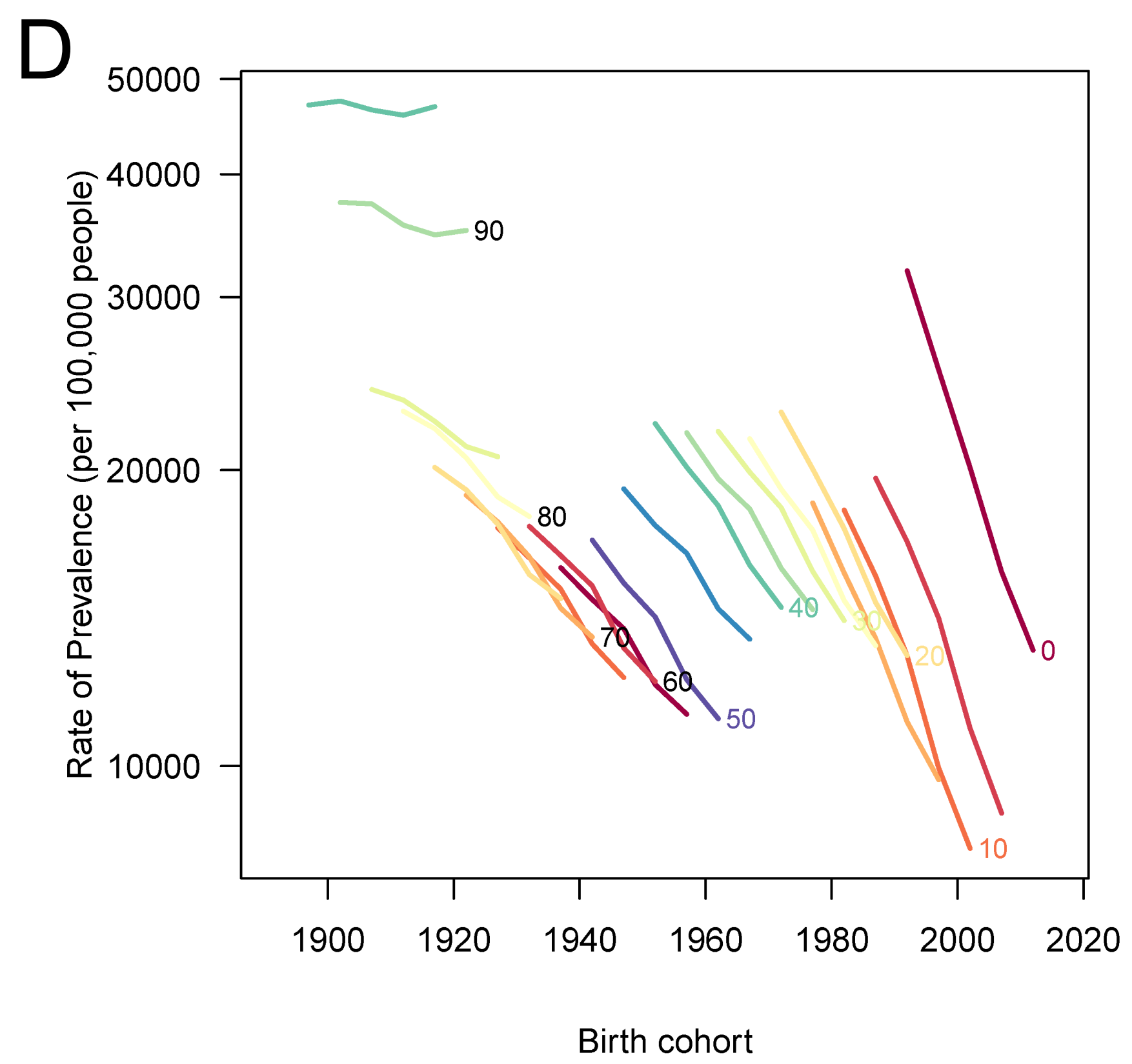

Supplement: SUPPLEMENTARY FIGURE S4 — Age-period-cohort analysis of age-standardized prevalence rates of nutritional deficiency in China. (A) Age-specific prevalence rates across successive time periods (1990–2021), with each line connecting the prevalence rate of a specific age group in each period. (B) Age-specific prevalence rates across successive birth cohorts, with each line connecting the prevalence rate of individuals born in a specific cohort across age groups. (C) Period-specific prevalence rates across age groups, with each line representing the prevalence rate of a fixed age group over different time periods. (D) Cohort-specific prevalence rates across age groups, with each line depicting the prevalence rate of a fixed birth cohort across age groups. [file Data_Sheet_4.PDF]

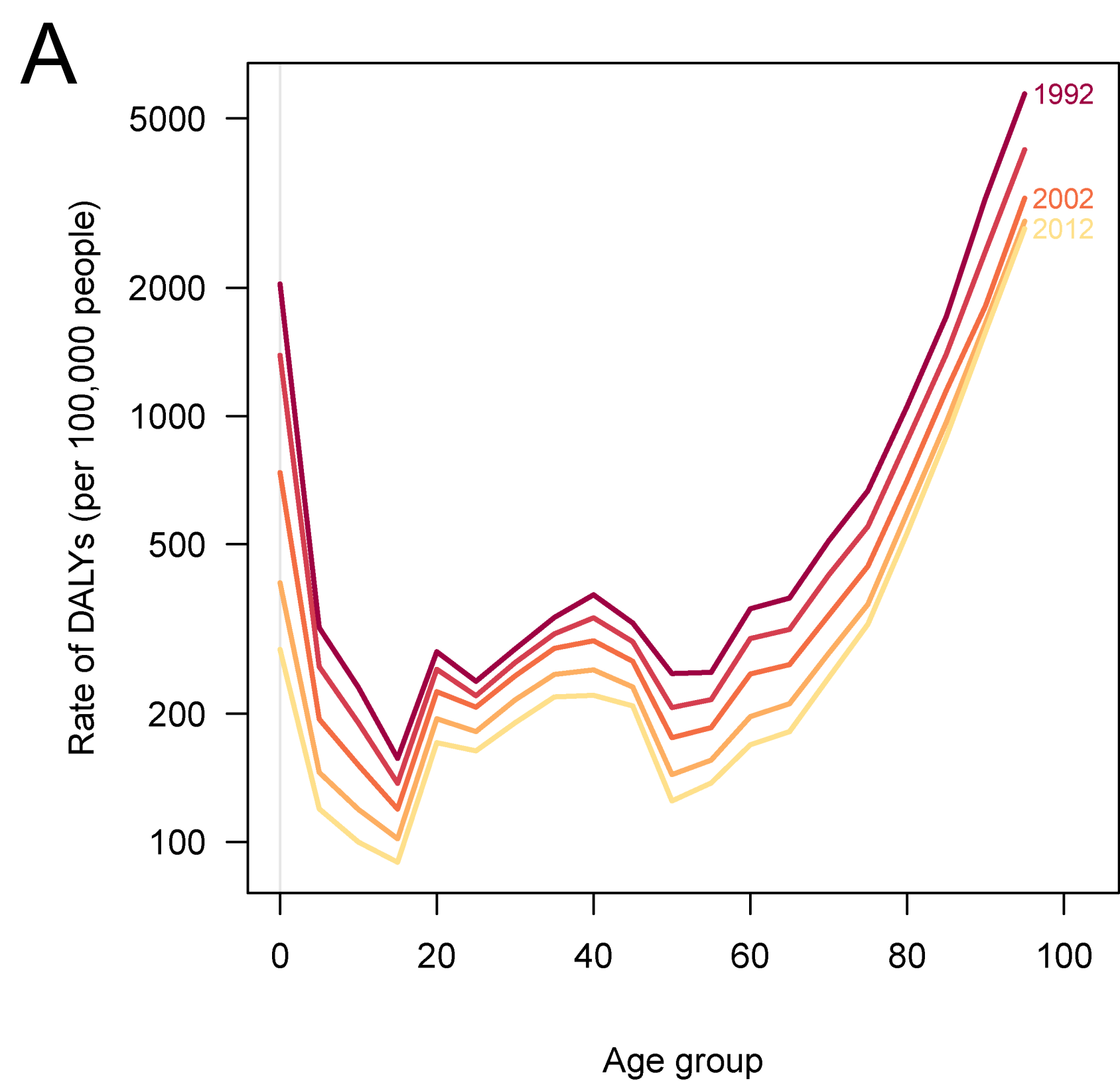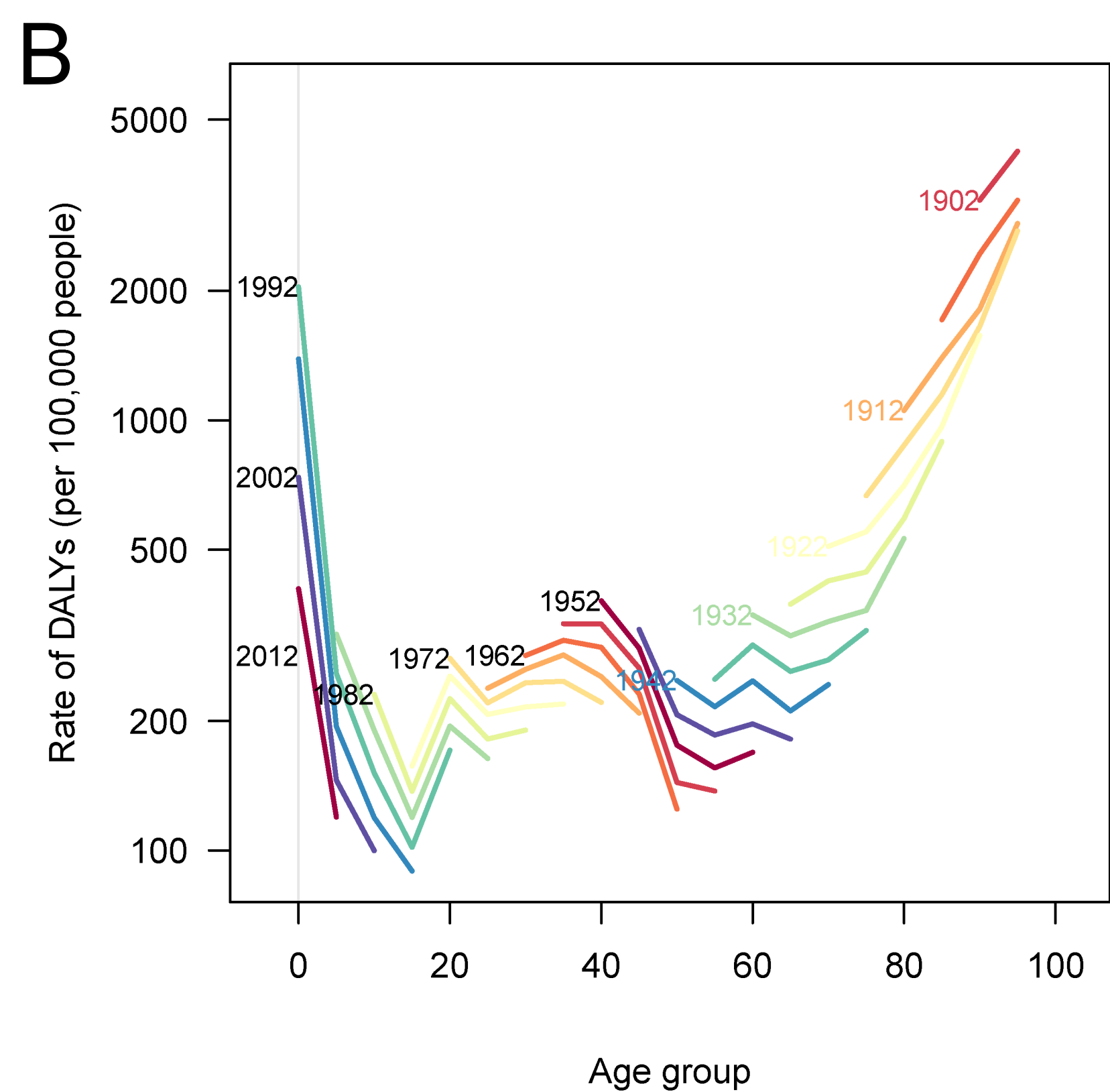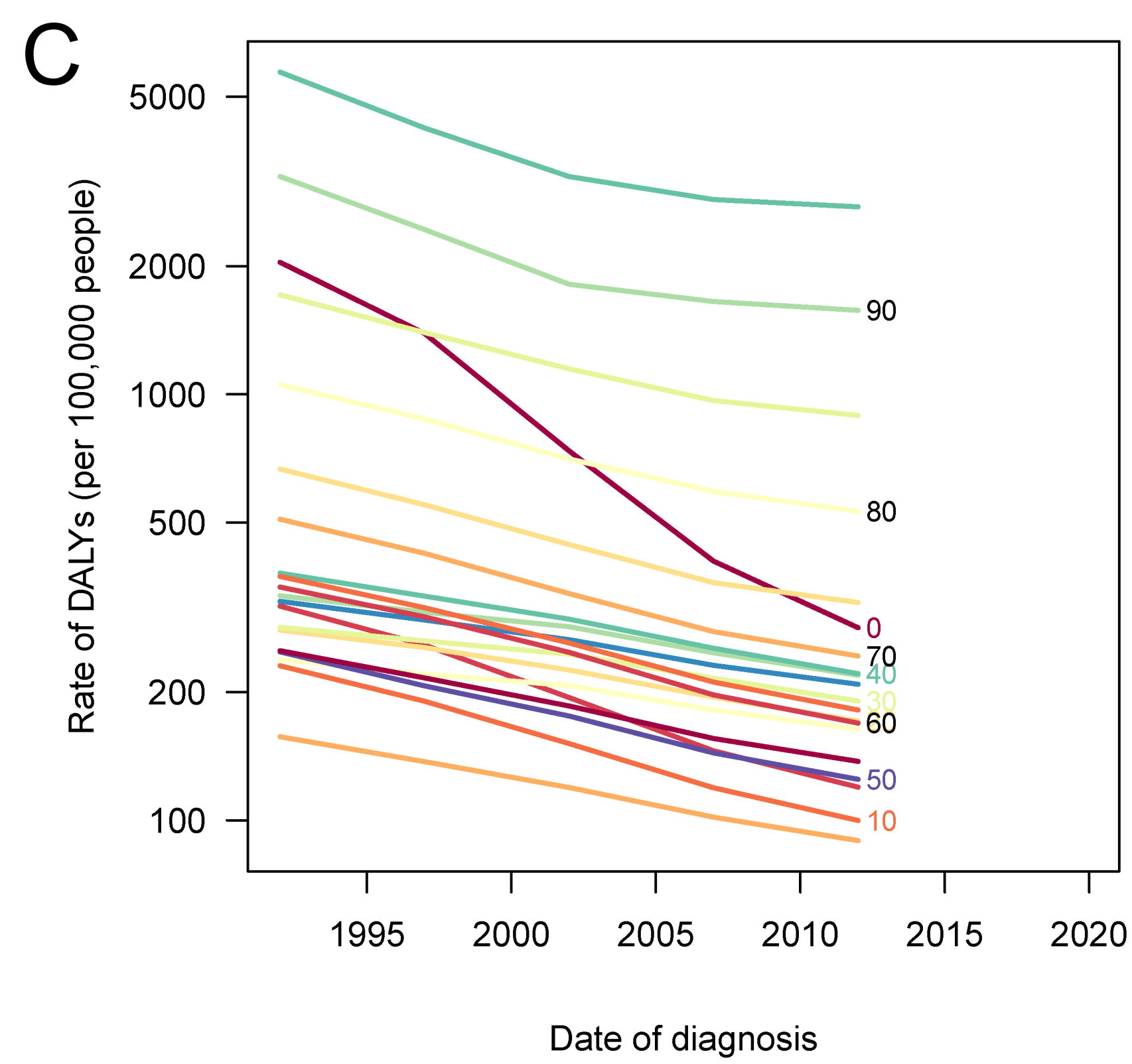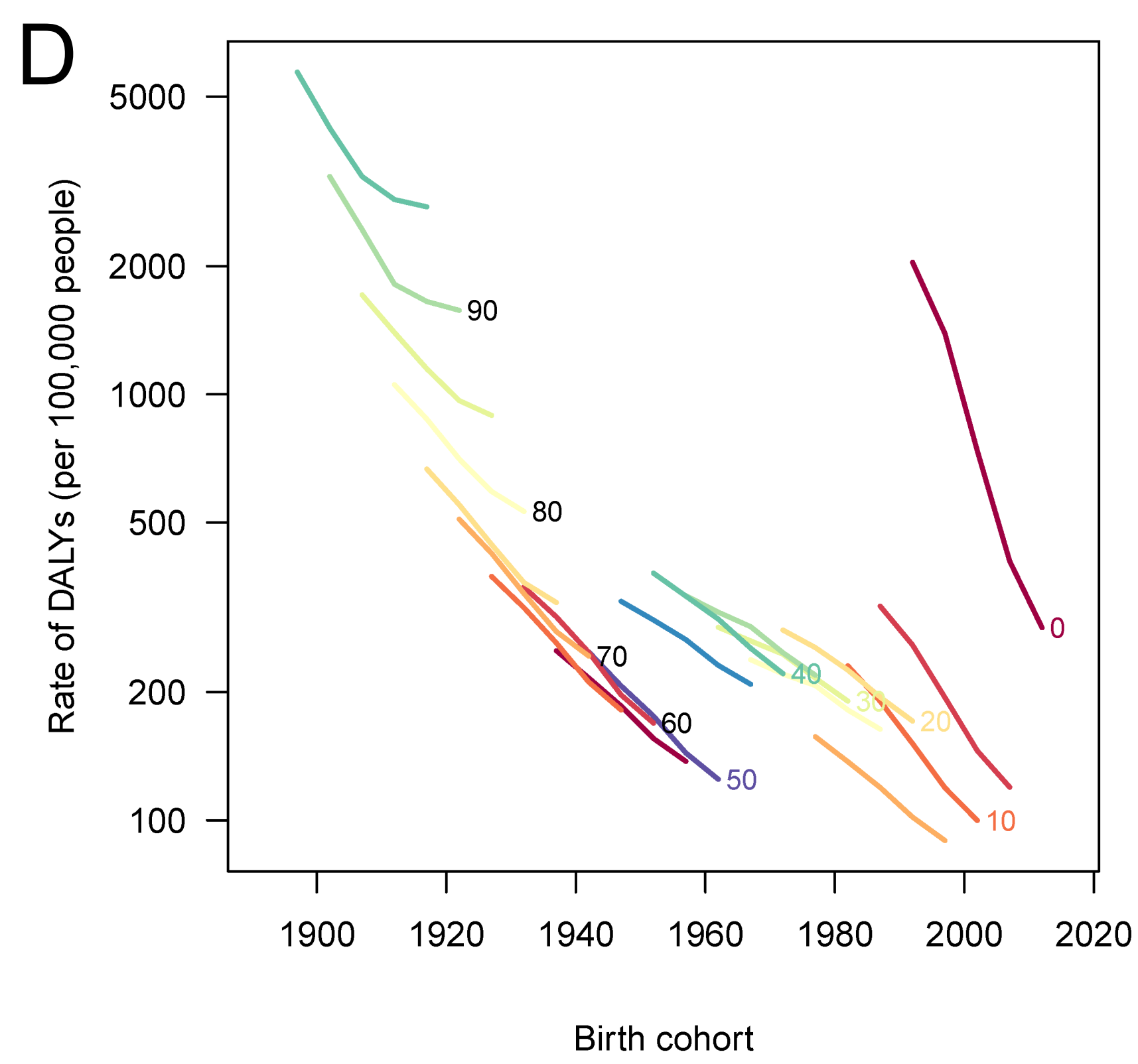

Supplement: SUPPLEMENTARY FIGURE S5 — Age-period-cohort analysis of DALYs due to nutritional deficiencies in China, 1990–2021. (A) Age-specific DALY rates across different time periods, where each line represents a 5-year period and illustrates changes in DALY rates across age groups. (B) Age-specific DALY rates across different birth cohorts, with each line indicating a 5-year birth cohort and showing variations in DALY rates by age within that cohort. (C) Period-specific DALY rates across age groups, where each line represents a 5-year age group and displays changes in DALY rates over successive time periods. (D) Birth cohort-specific DALY rates across age groups, with each line representing a 5-year age group and showing changes in DALY rates across different birth cohorts. DALYs, disability-adjusted life years. [file Data_Sheet_5.PDF]
