# Supplementary material for: Burden, trends, and projections of nutritional deficiencies in China from 1990 to 2030
Source: Front Nutr. 2025 Sep 4;12:1643869. doi: 10.3389/fnut.2025.1643869 (PMC12444020; doi:10.3389/fnut.2025.1643869)
Supplement: Supplementary file 6 [file Table_1.DOCX]

Table S1. Incidence, prevalence, and mortality for different type of nutritional deficiencies in China, 2021, with trends in ASRs per 100,000 population from 1990 to 2021

|  | Incidence | | | Prevalence | | | Deaths | | |
| --- | --- | --- | --- | --- | --- | --- | --- | --- | --- |
| Type of malnutrition | No, in thousands | Age-standardized rate per 100,000 | Percentage change from 1990 to 2021 | No, in thousands | Age-standardized rate per 100,000 | Percentage change from 1990 to 2021 | No, in thousands | Age-standardized rate per 100,000 | Percentage change from 1990 to 2021 |
| Protein-energy malnutrition | 22019.9 (17990.4, 26707.2) | 1387.9 (1133.8, 1713.4) | 13.1 (3.4, 24.6) | 21525.5 (18105.5, 25711.7) | 1354.9 (1151.7, 1623.8) | 1.9 (-5.9, 9.1) | 12.5 (10.4, 14.7) | 0.9 (0.8, 1.1) | -82.6 (-85.6, -78.9) |
| Vitamin A deficiency | 23194.5 (20122.6, 26881.3) | 1951 (1673, 2272) | -81.2 (-84.7, -76.3) | 23158.5 (20089.4, 26846.7) | 1947.5 (1669.7, 2268.7) | -81.2 (-84.7, -76.3) | NA | NA | NA |
| Dietary iron deficiency | NA | NA | NA | 79591.1 (76187.7, 82382.2) | 5313.2 (5103.5, 5502.8) | -54.7 (-56.0, -53.6) | NA | NA | NA |
| lodine deficiency | 789.1 (630.6, 963) | 70 (55.2, 85.7) | 4.5 (-4.8, 15.2) | 27503.5 (21598.2, 34010.5) | 1648.4 (1299.4, 2030.6) | 13 (2.7, 23.8) | NA | NA | NA |
| Other nutritional deficiencies | NA | NA | NA | NA | NA | NA | 3.2 (2.5, 4.1) | 0.2 (0.2, 0.3) | -23 (-41, 0.9) |

Values in parentheses indicate 95% UIs, estimated using Monte Carlo simulations. NA in the table represents that this part of data is not provided in the database. Abbreviations: ASRs, Age-standardized rates; UI, uncertainty interval.
