# Supplementary material for: Burden, trends, and projections of nutritional deficiencies in China from 1990 to 2030
Source: Front Nutr. 2025 Sep 4;12:1643869. doi: 10.3389/fnut.2025.1643869 (PMC12444020; doi:10.3389/fnut.2025.1643869)
Supplement: Supplementary file 7 [file Table_2.DOCX]

Table S2. DALYs, YLDs, and YLLs for different type of nutritional deficiencies in China, 2021, with trends in ASRs per 100,000 population from 1990 to 2021

|  | DALYs | | | YLDs | | | YLLs | | |
| --- | --- | --- | --- | --- | --- | --- | --- | --- | --- |
| Cancer type | No, in thousands | Age-standardized rate per 100,000 | Percentage change from 1990 to 2021 | No, in thousands | Age-standardized rate per 100,000 | Percentage change from 1990 to 2021 | No, in thousands | Age-standardized rate per 100,000 | Percentage change from 1990 to 2021 |
| Protein-energy malnutrition | 213.7 (180.4, 253.6) | 17.5 (14.9, 20.3) | -92.8 (-94.3, -91.2) | 9.1 (2.7, 28.0) | 0.7 (0.3, 1.8) | -93.9 (-97.7, -84) | 204.6 (173.7, 238.5) | 16.9 (14.4, 19.5) | -92.8 (-94.3, -91.0) |
| Vitamin A deficiency | 39.1 (24.9, 57.2) | 3.4 (2.1, 5.1) | -67.7 (-74.6, -59.7) | 39.1 (24.9, 57.2) | 3.4 (2.1, 5.1) | -67.7 (-74.6, -59.7) | NA | NA | NA |
| Dietary iron deficiency | 1689.2 (1119.3, 2446.0) | 115.3 (76.4, 166.4) | -59.7 (-61.7, -57.6) | 1689.2 (1119.3, 2446.0) | 115.3 (76.4, 166.4) | -59.7 (-61.7, -57.6) | NA | NA | NA |
| lodine deficiency | 293.9 (136.9, 564.7) | 17.7 (8.2, 33.9) | -7.8 (-31.6, 10.4) | 293.9 (136.9, 564.7) | 17.7 (8.2, 33.9) | -7.8 (-31.6, 10.4) | NA | NA | NA |
| Other nutritional deficiencies | 64 (50.7, 79.1) | 5.3 (4.3, 6.5) | -63.8 (-73.7, -50.3) | 2.8 (0.6, 9.8) | 0.2 (0, 0.6) | -93 (-98.2, -75.6) | 61.2 (48.9, 74.8) | 5.2 (4.2, 6.2) | -58.2 (-69.5, -41.5) |

Values in parentheses indicate 95% UIs, estimated using Monte Carlo simulations. NA in the table represents that this part of data is not provided in the database. Abbreviations: DALYs, disability-adjusted life-years; YLDs, years lived with disability; YLLs, years of life lost; ASRs, Age-standardized rates; UI, uncertainty interval.
