# Supplementary material for: Burden, trends, and projections of nutritional deficiencies in China from 1990 to 2030
Source: Front Nutr. 2025 Sep 4;12:1643869. doi: 10.3389/fnut.2025.1643869 (PMC12444020; doi:10.3389/fnut.2025.1643869)
Supplement: Supplementary file 8 [file Table_3.DOCX]

Table S1. Joinpoint regression analysis of trends in age-standardized incidence, prevalence, mortality rates (per 100,000) by sex for nutritional deficiencies in China, 1990-2021.

|  | ASIR |  |  | ASPR |  |  | ASMR |  |  |
| --- | --- | --- | --- | --- | --- | --- | --- | --- | --- |
| Gender | Period | APC (95% CI) | AAPC (95% CI) | Period | APC (95% CI) | AAPC (95% CI) | Period | APC (95% CI) | AAPC (95% CI) |
| Both | 1990-2000 | -3.72 (-4.34 - -3.55) ^*^ | -3.97 (-4.03 - -3.92) ^*^ | 1990-1995 | -2.16 (-2.30 - -1.89) ^*^ | -2.73 (-2.75 - -2.71) ^*^ | 1990-1995 | -5.00 (-6.00 - -3.70) ^*^ | -4.97 (-5.09 - -4.86) ^*^ |
|  | 2000-2005 | -3.30 (-5.43 - -2.81) ^*^ |  | 1995-2000 | -2.71 (-2.98 - -2.58) ^*^ |  | 1995-2007 | -8.77 (-9.00 - -8.57) ^*^ |  |
|  | 2005-2010 | -5.25 (-5.69 - -0.66) ^*^ |  | 2000-2005 | -2.33 (-2.45 - -2.12) ^*^ |  | 2007-2021 | -1.58 (-1.82 - -1.37) ^*^ |  |
|  | 2010-2015 | -0.77 (-6.39 - -0.38) ^*^ |  | 2005-2010 | -3.89 (-4.01 - -3.75) ^*^ |  |  |  |  |
|  | 2015-2021 | -6.46 (-6.84 - -6.07) ^*^ |  | 2010-2015 | -1.81 (-1.98 - -1.67) ^*^ |  |  |  |  |
|  |  |  |  | 2015-2021 | -3.34 (-3.47 - -3.20) ^*^ |  |  |  |  |
| Female | 1990-1995 | -1.44 (-1.66 - -1.12) ^*^ | -2.97 (-3.00 - -2.94) ^*^ | 1990-1995 | -1.04 (-1.16 - -0.92) ^*^ | -1.78 (-1.80 - -1.77) ^*^ | 1990-1995 | -5.35 (-6.33 - -4.44) ^*^ | -6.06 (-6.20 - -5.92) ^*^ |
|  | 1995-2002 | -2.33 (-2.45 - -2.15) ^*^ |  | 1995-2000 | -1.75 (-1.91 - -1.65) ^*^ |  | 1995-2007 | -10.23 (-10.51 - -9.99) ^*^ |  |
|  | 2002-2005 | -3.12 (-3.81 - -2.64) ^*^ |  | 2000-2005 | -1.48 (-1.55 - -1.34) ^*^ |  | 2007-2013 | -3.64 (-9.17 - -3.13) ^*^ |  |
|  | 2005-2010 | -4.53 (-4.72 - -4.37) ^*^ |  | 2005-2010 | -2.86 (-2.93 - -2.78) ^*^ |  | 2013-2021 | -1.81 (-2.52 - -0.10) ^*^ |  |
|  | 2010-2015 | -1.03 (-1.18 - -0.89) ^*^ |  | 2010-2015 | -1.34 (-1.45 - -1.24) ^*^ |  |  |  |  |
|  | 2015-2021 | -5.17 (-5.31 - -5.04) ^*^ |  | 2015-2021 | -2.15 (-2.24 - -2.05) ^*^ |  |  |  |  |
| Male | 1990-2000 | -4.95 (-5.13 - -4.81) ^*^ | -4.62 (-4.68 - -4.58) ^*^ | 1990-2005 | -3.47 (-3.52 - -3.41) ^*^ | -3.82 (-3.87 - -3.79) ^*^ | 1990-1995 | -4.13 (-5.03 - -2.77) ^*^ | -3.81 (-4.03 - -3.65) ^*^ |
|  | 2000-2005 | -3.60 (-4.01 - -2.92) ^*^ |  | 2005-2010 | -5.18 (-5.44 - -4.84) ^*^ |  | 1995-2006 | -7.22 (-7.47 - -7.02) ^*^ |  |
|  | 2005-2010 | -5.86 (-6.25 - -5.51) ^*^ |  | 2010-2015 | -2.26 (-2.58 - -1.98) ^*^ |  | 2006-2017 | -0.30 (-0.55 - 0.03) |  |
|  | 2010-2015 | -0.25 (-0.55 - 0.06) |  | 2015-2019 | -5.48 (-6.02 - -4.94) ^*^ |  | 2017-2021 | -3.34 (-7.19 - -1.63) ^*^ |  |
|  | 2015-2019 | -8.32 (-8.93 - -7.96) ^*^ |  | 2019-2021 | -3.53 (-4.61 - -2.93) ^*^ |  |  |  |  |
|  | 2019-2021 | -5.57 (-6.75 - -4.66) ^*^ |  |  |  |  |  |  |  |

Abbreviations: AAPC, average annual percent change presented for full period; APC, annual percent change; CI, confidence interval. ^*^, *p* <0.05 (permutation test).
