# Supplementary material for: Burden, trends, and projections of nutritional deficiencies in China from 1990 to 2030
Source: Front Nutr. 2025 Sep 4;12:1643869. doi: 10.3389/fnut.2025.1643869 (PMC12444020; doi:10.3389/fnut.2025.1643869)
Supplement: Supplementary file 10 [file Table_5.DOCX]

Table S5. Joinpoint regression analysis of trends in age-standardized prevalence, DALY, and YLD rates (per 100,000) by sex for dietary iron deficiency in China, 1990-2021.

|  | ASPR |  |  | DALYs |  |  | YLDs |  |  |
| --- | --- | --- | --- | --- | --- | --- | --- | --- | --- |
| Gender | Period | APC (95% CI) | AAPC (95% CI) | Period | APC (95% CI) | AAPC (95% CI) | Period | APC (95% CI) | AAPC (95% CI) |
| Both | 1990-1996 | -1.72 (-1.80 - -1.63) ^*^ | -2.52 (-2.54 - -2.50) ^*^ | 1990-1997 | -1.94 (-2.02 - -1.86) ^*^ | -2.89 (-2.90 - -2.87) ^*^ | 1990-1997 | -1.94 (-2.02 - -1.86) ^*^ | -2.89 (-2.90 - -2.87) ^*^ |
|  | 1996-2001 | -2.50 (-2.61 - -2.40) ^*^ |  | 1997-2001 | -2.89 (-3.10 - -2.72) ^*^ |  | 1997-2001 | -2.89 (-3.10 - -2.72) ^*^ |  |
|  | 2001-2010 | -3.51 (-3.55 - -3.47) ^*^ |  | 2001-2010 | -4.11 (-4.16 - -4.07) ^*^ |  | 2001-2010 | -4.11 (-4.16 - -4.07) ^*^ |  |
|  | 2010-2018 | -2.34 (-2.42 - -2.28) ^*^ |  | 2010-2018 | -2.69 (-2.77 - -2.64) ^*^ |  | 2010-2018 | -2.69 (-2.77 - -2.64) ^*^ |  |
|  | 2018-2021 | -1.64 (-1.93 - -1.24) ^*^ |  | 2018-2021 | -1.86 (-2.13 - -1.45) ^*^ |  | 2018-2021 | -1.86 (-2.13 - -1.45) ^*^ |  |
| Female | 1990-1996 | -1.25 (-1.33 - -1.17) ^*^ | -1.71 (-1.73 - -1.70) ^*^ | 1990-1996 | -0.94 (-1.02 - -0.85) ^*^ | -1.87 (-1.88 - -1.85) ^*^ | 1990-1996 | -0.94 (-1.02 - -0.85) ^*^ | -1.87 (-1.88 - -1.85) ^*^ |
|  | 1996-2001 | -1.90 (-1.99 - -1.77) ^*^ |  | 1996-2001 | -1.77 (-1.90 - -1.66) ^*^ |  | 1996-2001 | -1.77 (-1.90 - -1.66) ^*^ |  |
|  | 2001-2010 | -2.31 (-2.36 - -2.28) ^*^ |  | 2001-2010 | -2.71 (-2.77 - -2.67) ^*^ |  | 2001-2010 | -2.71 (-2.77 - -2.67) ^*^ |  |
|  | 2010-2018 | -1.53 (-1.61 - -1.48) ^*^ |  | 2010-2018 | -1.90 (-1.98 - -1.85) ^*^ |  | 2010-2018 | -1.90 (-1.98 - -1.85) ^*^ |  |
|  | 2018-2021 | -0.99 (-1.24 - -0.66) ^*^ |  | 2018-2021 | -1.22 (-1.45 - -0.86) ^*^ |  | 2018-2021 | -1.22 (-1.45 - -0.86) ^*^ |  |
| Male | 1990-1996 | -2.36 (-2.47 - -2.23) ^*^ | -3.82 (-3.85 - -3.80) ^*^ | 1990-1997 | -3.35 (-3.45 - -3.23) ^*^ | -5.05 (-5.08 - -5.03) ^*^ | 1990-1997 | -3.35 (-3.45 - -3.23) ^*^ | -5.05 (-5.08 - -5.03) ^*^ |
|  | 1996-2000 | -3.26 (-3.46 - -3.04) ^*^ |  | 1997-2001 | -4.68 (-4.88 - -4.42) ^*^ |  | 1997-2001 | -4.68 (-4.88 - -4.42) ^*^ |  |
|  | 2000-2003 | -4.60 (-4.92 - -4.26) ^*^ |  | 2001-2005 | -6.86 (-7.05 - -6.53) ^*^ |  | 2001-2005 | -6.86 (-7.05 - -6.53) ^*^ |  |
|  | 2003-2010 | -5.53 (-5.65 - -5.46) ^*^ |  | 2005-2010 | -7.45 (-7.63 - -7.32) ^*^ |  | 2005-2010 | -7.45 (-7.63 - -7.32) ^*^ |  |
|  | 2010-2018 | -3.79 (-3.91 - -3.71) ^*^ |  | 2010-2018 | -4.84 (-4.94 - -4.76) ^*^ |  | 2010-2018 | -4.84 (-4.94 - -4.76) ^*^ |  |
|  | 2018-2021 | -2.74 (-3.15 - -2.19) ^*^ |  | 2018-2021 | -3.58 (-3.94 - -2.97) ^*^ |  | 2018-2021 | -3.58 (-3.94 - -2.97) ^*^ |  |

Abbreviations: AAPC, average annual percent change presented for full period; APC, annual percent change; CI, confidence interval; ^*^, *p* <0.05 (permutation test).
