# Supplementary material for: Burden, trends, and projections of nutritional deficiencies in China from 1990 to 2030
Source: Front Nutr. 2025 Sep 4;12:1643869. doi: 10.3389/fnut.2025.1643869 (PMC12444020; doi:10.3389/fnut.2025.1643869)
Supplement: Supplementary file 11 [file Table_6.DOCX]

Table S6. Joinpoint regression analysis of trends in age-standardized incidence, and prevalence rates (per 100,000) by sex for iodine deficiency in China, 1990-2021.

|  | ASIR |  |  | ASPR |  |  |
| --- | --- | --- | --- | --- | --- | --- |
| Gender | Period | APC (95% CI) | AAPC (95% CI) | Period | APC (95% CI) | AAPC (95% CI) |
| Both | 1990-2000 | 0.70 (0.56 - 0.85) ^*^ | 0.09 (0.04 - 0.14) ^*^ | 1990-2000 | 0.94 (0.79 - 1.09) ^*^ | 0.35 (0.30 - 0.40) ^*^ |
|  | 2000-2005 | 5.46 (5.21 - 5.74) ^*^ |  | 2000-2005 | 6.33 (6.05 - 6.62) ^*^ |  |
|  | 2005-2015 | -2.23 (-2.48 - -2.09) ^*^ |  | 2005-2015 | -2.13 (-2.40 - -1.98) ^*^ |  |
|  | 2015-2021 | -1.38 (-1.71 - -0.74) ^*^ |  | 2015-2021 | -1.30 (-1.64 - -0.54) ^*^ |  |
| Female | 1990-1994 | 1.47 (0.83 - 2.59) ^*^ | 0.56 (0.50 - 0.62) ^*^ | 1990-1994 | 1.64 (1.00 - 2.87) ^*^ | 0.77 (0.69 - 0.83) ^*^ |
|  | 1994-2000 | 0.46 (-0.11 - 5.64) |  | 1994-2000 | 0.75 (0.12 - 6.64) ^*^ |  |
|  | 2000-2005 | 5.91 (-2.10 - 6.24) |  | 2000-2005 | 6.71 (-2.29 - 7.11) |  |
|  | 2005-2010 | -2.24 (-2.79 - -0.96) ^*^ |  | 2005-2010 | -2.20 (-2.85 - -0.46) ^*^ |  |
|  | 2010-2021 | -0.79 (-0.96 - -0.60) ^*^ |  | 2010-2021 | -0.78 (-1.13 - -0.51) ^*^ |  |
| Male | 1990-1995 | 0.94 (0.65 - 1.64) ^*^ | -0.50 (-0.55 - -0.45) ^*^ | 1990-2000 | 0.71 (0.57 - 0.86) ^*^ | -0.32 (-0.38 - -0.27) ^*^ |
|  | 1995-2000 | 0.06 (-0.39 - 0.35) |  | 2000-2005 | 6.04 (5.73 - 6.33) ^*^ |  |
|  | 2000-2004 | 5.90 (5.53 - 6.25) ^*^ |  | 2005-2010 | -2.58 (-2.94 - -2.03) ^*^ |  |
|  | 2004-2007 | -0.11 (-0.77 - 0.27) |  | 2010-2018 | -3.73 (-4.22 - -3.53) ^*^ |  |
|  | 2007-2018 | -3.54 (-3.66 - -3.44) ^*^ |  | 2018-2021 | -1.00 (-1.90 - 0.50) |  |
|  | 2018-2021 | -1.17 (-1.86 - -0.04) ^*^ |  |  |  |  |

Abbreviations: AAPC, average annual percent change presented for full period; APC, annual percent change; CI, confidence interval. ^*^, *p* <0.05 (permutation test).
