# Supplementary material for: Burden, trends, and projections of nutritional deficiencies in China from 1990 to 2030
Source: Front Nutr. 2025 Sep 4;12:1643869. doi: 10.3389/fnut.2025.1643869 (PMC12444020; doi:10.3389/fnut.2025.1643869)
Supplement: Supplementary file 12 [file Table_7.DOCX]

Table S4. Joinpoint regression analysis of trends in age-standardized DALY, and YLD rates (per 100,000) by sex for iodine deficiency in China, 1990-2021.

|  | DALYs |  |  | YLDs |  |  |
| --- | --- | --- | --- | --- | --- | --- |
| Gender | Period | APC (95% CI) | AAPC (95% CI) | Period | APC (95% CI) | AAPC (95% CI) |
| Both | 1990-2000 | -0.83 (-0.96 - -0.71) ^*^ | -0.25 (-0.29 - -0.20) ^*^ | 1990-2000 | -0.83 (-0.96 - -0.71) ^*^ | -0.25 (-0.29 - -0.20) ^*^ |
|  | 2000-2005 | 6.24 (5.98 - 6.51) ^*^ |  | 2000-2005 | 6.24 (5.98 - 6.51) ^*^ |  |
|  | 2005-2015 | -2.14 (-2.40 - -2.00) ^*^ |  | 2005-2015 | -2.14 (-2.40 - -2.00) ^*^ |  |
|  | 2015-2021 | -1.32 (-1.65 - -0.62) ^*^ |  | 2015-2021 | -1.32 (-1.65 - -0.62) ^*^ |  |
| Female | 1990-2000 | -0.45 (-0.58 - -0.32) ^*^ | 0.23 (0.19 - 0.28) ^*^ | 1990-2000 | -0.45 (-0.58 - -0.32) ^*^ | 0.23 (0.19 - 0.28) ^*^ |
|  | 2000-2005 | 6.49 (6.19 - 6.79) ^*^ |  | 2000-2005 | 6.49 (6.19 - 6.79) ^*^ |  |
|  | 2005-2010 | -2.16 (-2.82 - -1.77) ^*^ |  | 2005-2010 | -2.16 (-2.82 - -1.77) ^*^ |  |
|  | 2010-2021 | -0.80 (-0.94 - -0.60) ^*^ |  | 2010-2021 | -0.80 (-0.94 - -0.60) ^*^ |  |
| Male | 1990-2000 | -1.51 (-1.66 - -1.36) ^*^ | -1.06 (-1.14 - -1.01) ^*^ | 1990-2000 | -1.51 (-1.66 - -1.36) ^*^ | -1.06 (-1.14 - -1.01) ^*^ |
|  | 2000-2004 | 6.49 (6.05 - 6.97) ^*^ |  | 2000-2004 | 6.49 (6.05 - 6.97) ^*^ |  |
|  | 2004-2007 | 0.18 (-0.81 - 1.06) |  | 2004-2007 | 0.18 (-0.81 - 1.06) |  |
|  | 2007-2018 | -3.57 (-3.80 - -3.43) ^*^ |  | 2007-2018 | -3.57 (-3.80 - -3.43) ^*^ |  |
|  | 2018-2021 | -1.23 (-2.33 - 0.27) |  | 2018-2021 | -1.23 (-2.33 - 0.27) |  |

Abbreviations: DALYs, disability-adjusted life years; YLDs, years lived with disability; YLLs, years of life lost; AAPC, average annual percent change presented for full period; APC, annual percent change; CI, confidence interval. ^*^, *p* <0.05.
