# Supplementary material for: Burden, trends, and projections of nutritional deficiencies in China from 1990 to 2030
Source: Front Nutr. 2025 Sep 4;12:1643869. doi: 10.3389/fnut.2025.1643869 (PMC12444020; doi:10.3389/fnut.2025.1643869)
Supplement: Supplementary file 13 [file Table_8.DOCX]

Table S8. Joinpoint regression analysis of trends in age-standardized incidence, prevalence, mortality rates (per 100,000) by sex for other nutritional deficiencies in China, 1990-2021.

|  | ASMR |  |  | DALYs |  |  |
| --- | --- | --- | --- | --- | --- | --- |
| Gender | Period | APC (95% CI) | AAPC (95% CI) | Period | APC (95% CI) | AAPC (95% CI) |
| Both | 1990-1995 | -4.90 (-9.01 - -2.83) | -0.92 (-1.14 - -0.67) ^*^ | 1990-1995 | -6.43 (-9.24 - -4.97) ^*^ | -3.17 (-3.41 - -3.01) ^*^ |
|  | 1995-1999 | -0.90 (-2.55 - 11.94) |  | 1995-1999 | -3.28 (-4.57 - 6.00) |  |
|  | 1999-2004 | 10.81 (-3.24 - 13.02) |  | 1999-2004 | 6.66 (-6.07 - 8.08) |  |
|  | 2004-2021 | -2.96 (-3.82 - -2.58) |  | 2004-2012 | -7.51 (-8.28 - -3.22) ^*^ |  |
|  |  |  |  | 2012-2019 | -3.61 (-7.52 - -2.98) ^*^ |  |
|  |  |  |  | 2019-2021 | 1.33 (-3.08 - 3.57) |  |
| Female | 1990-1997 | -4.79 (-6.04 - -4.17) ^*^ | -1.75 (-1.92 - -1.59) ^*^ | 1990-1999 | -6.13 (-7.22 - -5.04) ^*^ | -4.25 (-4.54 - -4.09) ^*^ |
|  | 1997-2000 | -0.23 (-2.65 - 9.25) |  | 1999-2004 | 4.36 (-6.48 - 5.24) |  |
|  | 2000-2004 | 11.17 (-2.85 - 12.85) |  | 2004-2013 | -8.34 (-9.12 - 4.86) |  |
|  | 2004-2013 | -4.65 (-5.37 - -4.20) ^*^ |  | 2013-2019 | -3.78 (-9.03 - -3.09) ^*^ |  |
|  | 2013-2021 | -2.36 (-3.16 - -0.94) ^*^ |  | 2019-2021 | 1.19 (-3.49 - 4.18) |  |
| Male | 1990-1995 | -4.63 (-7.55 - -3.09) ^*^ | 0.40 (0.23 - 0.58) ^*^ | 1990-1995 | -6.57 (-9.10 - -5.19) ^*^ | -2.21 (-2.47 - -2.05) ^*^ |
|  | 1995-1999 | 2.07 (-0.83 - 7.93) |  | 1995-1999 | -0.58 (-4.49 - 2.05) |  |
|  | 1999-2004 | 13.34 (12.24 - 15.03) ^*^ |  | 1999-2004 | 8.60 (0.28 - 9.76) ^*^ |  |
|  | 2004-2021 | -2.02 (-2.27 - -1.82) ^*^ |  | 2004-2011 | -6.67 (-7.46 - 8.58) |  |
|  |  |  |  | 2011-2019 | -3.41 (-6.97 - -2.93) ^*^ |  |
|  |  |  |  | 2019-2021 | 0.89 (-3.19 - 3.14) |  |

Abbreviations: AAPC, average annual percent change presented for full period; APC, annual percent change; CI, confidence interval. ^*^, *p* <0.05 (permutation test).
