# Supplementary material for: Burden, trends, and projections of nutritional deficiencies in China from 1990 to 2030
Source: Front Nutr. 2025 Sep 4;12:1643869. doi: 10.3389/fnut.2025.1643869 (PMC12444020; doi:10.3389/fnut.2025.1643869)
Supplement: Supplementary file 14 [file Table_9.DOCX]

Table S4. Joinpoint regression analysis of trends in age-standardized DALY, YLD, and YLL rates (per 100,000) by sex for other nutritional deficiencies in China, 1990-2021.

|  | DALYs |  |  | YLDs |  |  | YLLs |  |  |
| --- | --- | --- | --- | --- | --- | --- | --- | --- | --- |
| Gender | Period | APC (95% CI) | AAPC (95% CI) | Period | APC (95% CI) | AAPC (95% CI) | Period | APC (95% CI) | AAPC (95% CI) |
| Both | 1990-1996 | -3.68 (-4.14 - -3.18) ^*^ | -4.15 (-4.21 - -4.08) ^*^ | 1990-1996 | -2.16 (-2.27 - -2.08) ^*^ | -2.78 (-2.80 - -2.77) ^*^ | 1990-1995 | -5.63 (-6.69 - -3.94) ^*^ | -7.68 (-7.81 - -7.54) ^*^ |
|  | 1996-2008 | -5.79 (-6.23 - -3.79) ^*^ |  | 1996-2002 | -2.90 (-2.97 - -2.20) ^*^ |  | 1995-2000 | -9.44 (-10.61 - -8.11) ^*^ |  |
|  | 2008-2012 | -3.70 (-5.92 - -2.74) ^*^ |  | 2002-2005 | -3.19 (-4.01 - -2.98) ^*^ |  | 2000-2004 | -15.22 (-16.50 - -13.08) ^*^ |  |
|  | 2012-2021 | -2.43 (-2.78 - -1.52) ^*^ |  | 2005-2010 | -4.01 (-4.11 - -2.66) ^*^ |  | 2004-2007 | -12.60 (-14.13 - -7.05) ^*^ |  |
|  |  |  |  | 2010-2018 | -2.59 (-2.67 - -2.52) ^*^ |  | 2007-2012 | -7.06 (-8.06 - -2.54) ^*^ |  |
|  |  |  |  | 2018-2021 | -1.80 (-2.02 - -1.47) ^*^ |  | 2012-2021 | -2.87 (-3.60 - -2.16) ^*^ |  |
| Female | 1990-1992 | -2.04 (-2.99 - -1.39) ^*^ | -3.41 (-3.45 - -3.36) ^*^ | 1990-1996 | -1.20 (-1.28 - -1.13) ^*^ | -1.80 (-1.81 - -1.78) ^*^ | 1990-1998 | -7.27 (-7.94 - -6.63) ^*^ | -9.01 (-9.17 - -8.86) ^*^ |
|  | 1992-1997 | -3.90 (-4.53 - -3.61) ^*^ |  | 1996-2005 | -1.87 (-1.90 - -1.82) ^*^ |  | 1998-2007 | -15.79 (-16.30 - -15.34) ^*^ |  |
|  | 1997-2004 | -5.33 (-5.88 - -5.09) ^*^ |  | 2005-2010 | -2.79 (-2.88 - -2.71) ^*^ |  | 2007-2013 | -8.08 (-9.30 - -7.19) ^*^ |  |
|  | 2004-2010 | -3.97 (-4.42 - -3.34) ^*^ |  | 2010-2018 | -1.78 (-1.85 - -1.73) ^*^ |  | 2013-2021 | -3.32 (-4.11 - -2.38) ^*^ |  |
|  | 2010-2021 | -1.87 (-2.03 - -1.69) ^*^ |  | 2018-2021 | -1.17 (-1.39 - -0.86) ^*^ |  |  |  |  |
| Male | 1990-1994 | -3.93 (-4.68 - -2.73) ^*^ | -5.32 (-5.39 - -5.26) ^*^ | 1990-1996 | -3.53 (-3.64 - -3.42) ^*^ | -4.68 (-4.70 - -4.66) ^*^ | 1990-1994 | -4.74 (-5.94 - -1.99) ^*^ | -6.30 (-6.42 - -6.16) ^*^ |
|  | 1994-1999 | -5.45 (-7.37 - -4.92) ^*^ |  | 1996-2001 | -4.55 (-4.70 - -4.34) ^*^ |  | 1994-2000 | -7.48 (-8.53 - -6.89) ^*^ |  |
|  | 1999-2010 | -7.37 (-7.73 - -4.21) ^*^ |  | 2001-2005 | -5.42 (-5.70 - -5.21) ^*^ |  | 2000-2003 | -13.76 (-14.52 - -12.12) ^*^ |  |
|  | 2010-2021 | -3.68 (-3.96 - -3.41) ^*^ |  | 2005-2010 | -6.72 (-6.84 - -6.60) ^*^ |  | 2003-2007 | -10.74 (-11.34 - -6.35) ^*^ |  |
|  |  |  |  | 2010-2019 | -4.47 (-4.53 - -4.41) ^*^ |  | 2007-2012 | -5.08 (-5.92 - -3.71) ^*^ |  |
|  |  |  |  | 2019-2021 | -2.70 (-3.24 - -2.39) ^*^ |  | 2012-2021 | -2.17 (-2.61 - -1.65) ^*^ |  |

Abbreviations: DALYs, disability-adjusted life years; YLDs, years lived with disability; YLLs, years of life lost; AAPC, average annual percent change presented for full period; APC, annual percent change; CI, confidence interval. ^*^, *p* <0.05.
