# Supplementary material for: Burden, trends, and projections of nutritional deficiencies in China from 1990 to 2030
Source: Front Nutr. 2025 Sep 4;12:1643869. doi: 10.3389/fnut.2025.1643869 (PMC12444020; doi:10.3389/fnut.2025.1643869)
Supplement: Supplementary file 15 [file Table_10.DOCX]

Table S10. Joinpoint regression analysis of trends in age-standardized incidence, and prevalence rates (per 100,000) by sex for vitamin A deficiency in China, 1990-2021.

|  | ASIR |  |  | ASPR |  |  |
| --- | --- | --- | --- | --- | --- | --- |
| Gender | Period | APC (95% CI) | AAPC (95% CI) | Period | APC (95% CI) | AAPC (95% CI) |
| Both | 1990-1995 | -3.87 (-4.07 - -3.63) ^*^ | 0.09 (0.04 - 0.14) ^*^ | 1990-1995 | -3.87 (-4.07 - -3.63) | -5.26 (-5.29 - -5.23) ^*^ |
|  | 1995-2005 | -4.77 (-4.83 - -4.71) ^*^ |  | 1995-2005 | -4.77 (-4.83 - -4.71) |  |
|  | 2005-2010 | -7.05 (-7.16 - -6.95) ^*^ |  | 2005-2010 | -7.06 (-7.17 - -6.96) |  |
|  | 2010-2016 | -4.64 (-4.76 - -4.51) ^*^ |  | 2010-2016 | -4.64 (-4.77 - -4.52) |  |
|  | 2016-2021 | -6.51 (-6.71 - -6.31) ^*^ |  | 2016-2021 | -6.53 (-6.72 - -6.34) |  |
| Female | 1990-1995 | -1.56 (-1.76 - -1.38) ^*^ | -4.09 (-4.12 - -4.06) ^*^ | 1990-1995 | -1.57 (-1.75 - -1.39) ^*^ | -4.09 (-4.12 - -4.07) ^*^ |
|  | 1995-2001 | -3.16 (-3.29 - -3.03) ^*^ |  | 1995-2001 | -3.16 (-3.28 - -3.03) ^*^ |  |
|  | 2001-2005 | -4.34 (-4.52 - -4.14) ^*^ |  | 2001-2005 | -4.34 (-4.52 - -4.15) ^*^ |  |
|  | 2005-2010 | -6.16 (-6.30 - -6.04) ^*^ |  | 2005-2010 | -6.17 (-6.30 - -6.05) ^*^ |  |
|  | 2010-2016 | -3.87 (-4.03 - -3.73) ^*^ |  | 2010-2016 | -3.88 (-4.03 - -3.74) ^*^ |  |
|  | 2016-2021 | -5.64 (-5.89 - -5.38) ^*^ |  | 2016-2021 | -5.65 (-5.89 - -5.39) ^*^ |  |
| Male | 1990-1995 | -5.12 (-5.28 - -4.96) ^*^ | -6.13 (-6.15 - -6.10) ^*^ | 1990-1995 | -5.13 (-5.29 - -4.97) ^*^ | -6.13 (-6.16 - -6.11) ^*^ |
|  | 1995-2000 | -6.15 (-6.36 - -6.00) ^*^ |  | 1995-2000 | -6.14 (-6.36 - -6.00) ^*^ |  |
|  | 2000-2005 | -5.12 (-5.24 - -4.96) ^*^ |  | 2000-2005 | -5.12 (-5.24 - -4.96) ^*^ |  |
|  | 2005-2010 | -7.83 (-7.94 - -7.73) ^*^ |  | 2005-2010 | -7.85 (-7.95 - -7.74) ^*^ |  |
|  | 2010-2016 | -5.31 (-5.42 - -5.20) ^*^ |  | 2010-2016 | -5.31 (-5.43 - -5.20) ^*^ |  |
|  | 2016-2021 | -7.35 (-7.52 - -7.19) ^*^ |  | 2016-2021 | -7.37 (-7.54 - -7.21) ^*^ |  |

Abbreviations: AAPC, average annual percent change presented for full period; APC, annual percent change; CI, confidence interval. ^*^, *p* <0.05 (permutation test).
