# Supplementary material for: Burden, trends, and projections of nutritional deficiencies in China from 1990 to 2030
Source: Front Nutr. 2025 Sep 4;12:1643869. doi: 10.3389/fnut.2025.1643869 (PMC12444020; doi:10.3389/fnut.2025.1643869)
Supplement: Supplementary file 16 [file Table_11.DOCX]

Table S11. Joinpoint regression analysis of trends in age-standardized DALY, and YLD rates (per 100,000) by sex for vitamin A deficiency in China, 1990-2021.

|  | DALYs |  |  | YLDs |  |  |
| --- | --- | --- | --- | --- | --- | --- |
| Gender | Period | APC (95% CI) | AAPC (95% CI) | Period | APC (95% CI) | AAPC (95% CI) |
| Both | 1990-1994 | -4.84 (-5.19 - -4.14) ^*^ | -3.63 (-3.67 - -3.59) ^*^ | 1990-1994 | -4.84 (-5.19 - -4.14) | -3.63 (-3.67 - -3.59) ^*^ |
|  | 1994-1999 | -6.07 (-6.66 - -5.80) ^*^ |  | 1994-1999 | -6.07 (-6.66 - -5.80) |  |
|  | 1999-2007 | -5.03 (-5.26 - -4.86) ^*^ |  | 1999-2007 | -5.03 (-5.26 - -4.86) |  |
|  | 2007-2011 | -4.03 (-4.56 - -3.06) ^*^ |  | 2007-2011 | -4.03 (-4.56 - -3.06) |  |
|  | 2011-2015 | -1.82 (-2.39 - -1.03) ^*^ |  | 2011-2015 | -1.82 (-2.39 - -1.03) |  |
|  | 2015-2021 | 0.27 (0.03 - 0.59) ^*^ |  | 2015-2021 | 0.27 (0.03 - 0.59) |  |
| Female | 1990-1995 | -3.83 (-4.17 - -3.18) ^*^ | -2.89 (-2.92 - -2.84) ^*^ | 1990-1995 | -3.83 (-4.17 - -3.18) ^*^ | -2.89 (-2.92 - -2.84) ^*^ |
|  | 1995-1998 | -4.82 (-5.08 - -4.01) ^*^ |  | 1995-1998 | -4.82 (-5.08 - -4.01) ^*^ |  |
|  | 1998-2007 | -4.00 (-4.13 - -2.64) ^*^ |  | 1998-2007 | -4.00 (-4.13 - -2.64) ^*^ |  |
|  | 2007-2013 | -2.51 (-2.81 - -0.99) ^*^ |  | 2007-2013 | -2.51 (-2.81 - -0.99) ^*^ |  |
|  | 2013-2021 | -0.55 (-0.74 - -0.30) ^*^ |  | 2013-2021 | -0.55 (-0.74 - -0.30) ^*^ |  |
| Male | 1990-1994 | -5.47 (-5.87 - -4.86) ^*^ | -4.04 (-4.11 - -3.99) ^*^ | 1990-1994 | -5.47 (-5.87 - -4.86) ^*^ | -4.04 (-4.11 - -3.99) ^*^ |
|  | 1994-1999 | -7.03 (-7.75 - -6.69) ^*^ |  | 1994-1999 | -7.03 (-7.75 - -6.69) ^*^ |  |
|  | 1999-2010 | -5.60 (-5.73 - -5.47) ^*^ |  | 1999-2010 | -5.60 (-5.73 - -5.47) ^*^ |  |
|  | 2010-2014 | -2.52 (-4.60 - -2.05) ^*^ |  | 2010-2014 | -2.52 (-4.60 - -2.05) ^*^ |  |
|  | 2014-2019 | -0.03 (-1.51 - 0.42) |  | 2014-2019 | -0.03 (-1.51 - 0.42) |  |
|  | 2019-2021 | 2.40 (0.91 - 3.14) ^*^ |  | 2019-2021 | 2.40 (0.91 - 3.14) ^*^ |  |

Abbreviations: DALYs, disability-adjusted life years; YLDs, years lived with disability; YLLs, years of life lost; AAPC, average annual percent change presented for full period; APC, annual percent change; CI, confidence interval. ^*^, *p* <0.05.
